# Ionic‐Liquid Free and Flexible Transistors Made of 2D Material Inks

**DOI:** 10.1002/smll.202508360

**Published:** 2025-10-30

**Authors:** Liming Chen, Khaled Parvez, Francesco Nepa, Elisabetta Dimaggio, Chaochao Dun, Oliver Read, Jeffrey J. Urban, Gianluca Fiori, Cinzia Casiraghi

**Affiliations:** ^1^ Department of Chemistry University of Manchester Oxford Road Manchester M13 9PL United Kingdom; ^2^ Dipartimento di Ingegneria dell'Informazione University of Pisa Pisa 56122 Italy; ^3^ Lawrence Berkeley National Laboratory Berkeley CA 94720 USA

**Keywords:** flexible electronics, inkjet printing, interfacial self‐assembly, thin film transistors, 2D materials

## Abstract

The development of thin‐film transistors (TFTs) using 2D materials is crucial for enabling scalable, low‐cost, and flexible electronics. Currently, 2D TFTs with the highest performance have been achieved by using ionic‐liquid gating (ILG), a technique suited for proof‐of‐concept studies. However, ILG suffers from slow switching speeds, temperature sensitivity, poor long‐term stability, and integration challenges, making it unsuitable for practical use. Moreover, typical fabrication methods for 2D TFTs involve harsh conditions such as strong acids or high temperatures (>300 °C), limiting integration with flexible substrates. This work provides the first demonstration of an ILG‐free, all‐2D‐material TFT fabricated onto a flexible substrate. Water‐based graphene and hexagonal boron nitride (h‐BN) inks are printed to deposit the electrodes and dielectric layers, respectively. The MoS_2_ channel is produced via supramolecular interfacial self‐assembly, yielding uniform, monolayer‐rich films transferable to rigid and flexible substrates. The resulting TFTs operate below 3 V, exhibit negligible leakage current, and achieve field‐effect mobilities up to 0.46 cm^2^ V^−1^ s^−1^ (rising to 2.47 cm^2^ V^−1^ s^−1^ with silver electrodes) measured under ambient conditions, while maintaining excellent mechanical flexibility. This work establishes a low‐cost and scalable solution‐processable platform for flexible electronics based on 2D materials that match requirements for practical applications.

## Graphical abstract

This work demonstrates an all‐2D material thin‐film transistor (TFT) fabricated on a flexible substrate using inkjet‐printed graphene and h‐BN inks, along with a MoS_2_ film, assembled via supramolecular chemistry at the liquid interface. The device exhibits good mobility and on/off ratio, operates below 3 V under ambient conditions. This approach paves the way for the development of practical, application‐ready all‐2D flexible TFTs.

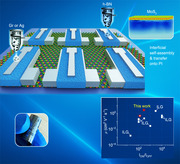

## 1 Introduction

2D materials are promising candidates for next‐generation TFTs due to their outstanding properties and compatibility with solution‐processed technologies by enabling the use of simple, cost‐effective, and low‐temperature approaches for device integration onto flexible substrates.^[^ ^1^, ^2^, ^3^ ^]^ In particular, semiconducting 2D transition metal dichalcogenides (TMDs), such as MoS_2_, WS_2_, MoSe_2_, have shown great promise as channels for TFTs,^[^ ^2^, ^4^, ^5^ ^]^ showing mobility (*µ*_FE_) up to 20 cm^2^ V^−1^ s^−1^.^[^ ^6^ ^]^ Despite these achievements, we still lack of 2D TFTs fabricated onto flexible substrates suitable for practical applications.

Designing a fabrication strategy for all‐2D TFTs compatible with flexible substrates remains challenging for several reasons: despite the semiconducting film being prepared by solution processing, fabrication of other functional layers (e.g., electrodes and dielectrics) usually needs at least one step under high‐vacuum conditions, such as thermal vapor deposition for the contacts,^[^ ^7^, ^8^ ^]^ or high temperature (T > 300 °C) processing for the oxide dielectrics (e.g., HfO_2_).^[^ ^6^, ^9^, ^10^ ^]^ Alternative dielectrics, such as quasi‐2D oxide^[^ ^11^ ^]^ and ion–Jacobson perovskite Sr_1.8_Bi_0.2_Nb_3_O_10_ nanosheets,^[^ ^12^ ^]^ have been explored. However, challenges remain in achieving uniform films to minimize leakage current and perovskite stability in air.^[^ ^11^ ^]^ Furthermore, the synthesis of Sr_1.8_Bi_0.2_Nb_3_O_10_ nanosheets requires calcination at 1200 °C for 10 h.

Because of the challenges in the dielectric film performance, most 2D material‐based TFTs reported so far make use of ILG, often in combination with pretreatment of the MoS_2_ film with bis(triflurolomethane)sulfonimide (TFSI), which is used to heal defects and therefore improve device mobility. For example, *µ*_FE_ up to 11 cm^2^ V^−1^ s^−1^ was obtained by 1‐ethyl‐3‐methylimidazolium bis(trifluoromethylsulfonyl)imide (EMIm TFSI) gating.^[^ ^7^ ^]^ TFSI is a hazardous nonoxidizing organic superacid, and its sensitivity toward moisture and temperature may lead to performance degradation in electronic devices,^[^ ^13^, ^14^, ^15^ ^]^ hence it is of crucial importance to develop alternative strategies for all‐2D TFT fabrication. The Langmuir‐Schaefer (LS) method has been recently used to demonstrate MoS_2_ TFT with mobility of ≈11 cm^2^ V^−1^ s^−1^ without using acid treatment and by using a processing temperature of 120 °C.^[^ ^7^, ^16^ ^]^ This remarkable achievement was, however, obtained by EMIm TFSI gating. Although ILG enhances the device performance, the low ionic mobility of EMIm TFSI (10^−6^ S m^−1^)^[^ ^17^ ^]^ at room temperature limits the device switching speed. Additionally, the electrochemical reaction between the IL and the channel material, along with the hygroscopic nature of the IL, can alter the device performance over time.^[^ ^18^, ^19^ ^]^ Most importantly, while ILG is a powerful technique for proof‐of‐concept demonstration, the slow switching speeds, temperature sensitivity, poor long‐term stability, and integration challenges make ILG unsuitable for practical use.

In addition to the above issues, the TFT fabrication onto a flexible substrate is more challenging as compared to rigid substrates because plastic and paper have considerably higher roughness and different wettability as well as chemical and thermal stabilities, as compared to silicon.^[^ ^2^ ^]^

In this work, we propose a novel approach for the fabrication of all‐2D material TFTs on rigid and flexible substrates without using ILG and high‐temperature (T >300 °C) processes. Liquid‐Liquid Interfacial (LLI) assembly has emerged as a promising approach for the fabrication of thin films made of solution‐processed nanosheets.^[^ ^20^, ^21^ ^]^ Here, we use a supramolecular‐based LLI technique able to provide rapid, scalable fabrication of uniform, free‐standing films with a thickness below 100 nm, made by single and few‐layer MoS_2_ nanosheets produced by electrochemical exfoliation (ECE). Unlike previous assembly methods for 2D materials, our approach is based on the use of perfluorinated molecules with low surface free energy (e.g., perfluorodecanethiol (PFT)), which are used to increase the hydrophobicity of the nanosheets, hence reducing the kinetic energy barrier to interfacial assembly.^[^ ^22^ ^]^ This enables to minimization of pin‐holes by achieving the production of highly dense thin films within seconds. The free‐standing nature of the films on the surface of water ensures effortless transfer onto any surface, including rigid, flexible, and complex geometries. This approach is used for the deposition of the semiconducting MoS_2_ film onto silicon and polyimide (PI), while graphene (or silver) and hexagonal Boron Nitride (h‐BN) inks are printed to fabricate the contacts and the dielectric layer, respectively. The compatibility of water‐based and inkjet printable h‐BN and graphene inks with channel materials and their low temperature (<300 °C) processability make this approach extremely attractive for TFT fabrication. The all 2D‐material, ILG‐free TFT onto PI operates at low voltage (≤3 V) and has mobility up to 2.47 and 0.46 cm^2^ V^−1^ s^−1^ with inkjet printed Ag and graphene electrodes, respectively, and shows good mechanical flexibility under ambient conditions.

This work demonstrates a simple, low‐cost, and scalable strategy toward the development of 2D materials‐based and flexible electronics suitable for practical applications.

## 2 Results and Discussion

### 2.1 Ink Preparation and Characterization

**Figure 1:**
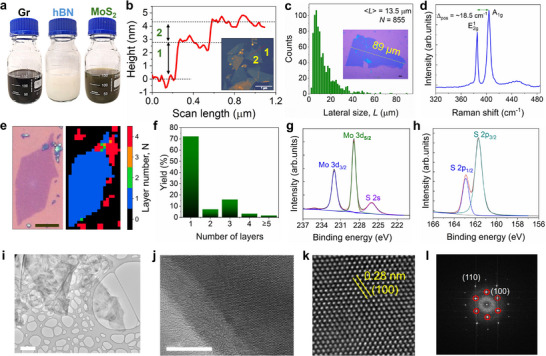
a) Photograph of graphene, hBN, and MoS_2_ inks. b) Profile of a representative nanosheet obtained by ECE using 0.5 mm TPA^+^ (inset: AFM image showing the selected nanosheet) measured by AFM. c) Statistical distribution of the lateral size of the MoS_2_ nanosheets using optical contrast taken on 855 individual nanosheets (*N*). Inset: optical microscope image of a large (89 µm) MoS_2_ nanosheet deposited on SiO_2_/Si substrate (scale bar = 5 µm). d) Representative Raman spectra of a single‐layer MoS_2_ nanosheet, showing the difference between the A_1g_ and $E_{2g}^{1}$ (i.e., Δ_pos_) peaks. e) Optical microscope image of a single‐layer MoS_2_ nanosheet (left panel) and corresponding Raman map (right panel), indicating uniform thickness distribution. Scale bar = 10 µm (left panel). f) Statistical thickness distribution of the MoS_2_ nanosheets obtained from the Raman spectra taken on more than 180 individual nanosheets. g) High resolution Mo 3d and h) S 2p XPS spectra of the MoS_2_ nanosheets. i) Representative low magnification; scale bar: 2 µm, and j) high resolution TEM image; scale bar: 10 nm. k) High angular dark field (HAADF) image of a single ECE MoS_2_ nanosheet showing (100) crystal plane with a lattice spacing of 0.28 nm. l) Corresponding fast Fourier transform (FFT) of MoS_2_. The red circles outline the six‐fold symmetric diffraction spots.

The device was made using a semiconducting MoS_2_ ink for the channel, graphene (or silver) ink for the contacts, and an h‐BN ink for the dielectric, **Figure** **1**a. The semiconducting nanosheets were produced by cathodic ECE of bulk MoS_2_ using quaternary ammonium cations.^[^ ^23^ ^]^ The exfoliation was performed with a two‐electrode system, where the bulk MoS_2_ crystal serves as a cathode, a Pt foil as an anode, and the electrolyte is made by dissolving tetrapropylammonium chloride (TPA^+^Cl^−^) in propylene carbonate (see Experimental sections for details and Figure S1, Supporting Information). Upon applying a voltage of −10 V to the MoS_2_ crystal, the TPA^+^ cations are intercalated into the bulk MoS_2_ crystal. A substantial volume expansion of the bulk crystal, which breaks into small pieces and suspends in the electrolyte (Figure S2a, Supporting Information), is observed, due to the size of the TPA^+^ cation (0.76 nm)^[^ ^24^ ^]^ being larger than the interlayer spacing of the MoS_2_ layers (0.615 nm). The greenish color of the MoS_2_ nanosheets dispersion (Figure S2b, Supporting Information) matches with the characteristic UV–vis absorption peaks at 419 nm and 452 nm, indicating direct transition from valence band to the conduction band, whereas peaks at 614 and 675 nm correspond to the direct bandgap transition occurring at K point of the Brillouin zone (Figure S3, Supporting Information), confirming the semiconducting nature of the as‐prepared MoS_2_ nanosheets.^[^ ^25^ ^]^ By comparing the results obtained using three different concentrations of the TPA^+^ ions (5 mm, 1 mm, and 0.5 mm), it was found that the largest and thinnest flakes were obtained with the smallest concentration. Figure 1b shows the height profile of a representative nanosheet, measured by Atomic Force Microscopy (AFM): its folded edge has a thickness of ≈1.50 nm, while the bottom layer is 2.80 nm thick, suggesting that trapped solvent can give an error margin >1 nm to the thickness. As a result, the apparent thickness of a single‐layer MoS_2_ can vary from 0.8 to 1.3 nm, as measured by AFM, in agreement with previous works.^[^ ^26^, ^27^, ^28^ ^]^ Lateral size analysis of more than 850 individual MoS_2_ nanosheets, performed using optical microscopy and ImageJ software, revealed an average nanosheet size of 13.5 µm (Figure 1c). The largest nanosheet was found to have a lateral size of ≈165 µm (Figure S3, Supporting Information). On the other hand, the MoS_2_ nanosheets obtained with 5 mm and 1 mm TPA^+^ cation concentration, revealed average nanosheet sizes of ≈7.50 and ≈7.63 µm, with the largest nanosheets of 39.8 and 28.6 µm, respectively (Figures S4 and S5, Supporting Information). These results clearly demonstrate that the size of the MoS_2_ nanosheets is dependent on the concentration of the TPA^+^ ions that intercalate into the MoS_2_ crystal: an excessive electrolyte concentration causes too many cations to intercalate between the MoS_2_ layers, leading to excessive charging and structural damage of the MoS_2_ nanosheets.^[^ ^29^ ^]^ Whereas, at low concentration, the number of ions that can intercalate is limited, causing a slow but gentle intercalation, hence giving rise to larger size MoS_2_ nanosheets. Based on these results, the MoS_2_ nanosheets obtained by ECE using 0.5 mm TPA^+^ were selected for device fabrication.

To further investigate the thickness and the quality of the nanosheets, more than 180 individual nanosheets were measured by Raman spectroscopy (Figure S6, Supporting Information). Figure 1d shows representative Raman spectra of selected nanosheets: their thickness is identified by measuring the difference in position between the $E_{2g}^{1}$ and A_1g_ peaks at 385.6 and 404.1 cm^−1^, respectively.^[^ ^30^, ^31^ ^]^ A nanosheet is a single layer when the peak difference (Δ_pos_ = A_1g_ – $E_{2g}^{1}$) is ≈18.5 cm^−1^. The full width at half‐maximum of the $E_{2g}^{1}\;$ and A_1g_ peaks are ≈6 and ≈4 cm^−1^, respectively, indicating high crystallinity.^[^ ^32^ ^]^ Figure 1e shows a Raman map of a flake of ≈35 µm lateral size: Δ_pos_ is ≈19 cm^−1^, all over the flake (Figure S7, Supporting Information), indicating uniform thickness and high crystallinity. The Raman spectroscopy results show that more than ≈80% of MoS_2_ nanosheets have a thickness below 3 layers, with ≈70% of MoS_2_ nanosheets being single‐layer (Figure 1f). Figure S8 (Supporting Information) shows the photoluminescence spectrum of a single‐layer MoS_2_, which is in agreement with results from previous works.^[^ ^10^, ^28^, ^33^ ^]^ The energy difference between the A and B peaks is ≈0.15 eV, which is in good agreement with the splitting energy of the valence band of single‐layer MoS_2_ at the K‐point by spin‐orbit interaction.^[^ ^34^, ^35^ ^]^

The MoS_2_ nanosheets were further characterised by X‐ray photoelectron spectroscopy (XPS). Figure 1g shows the Mo 3d spectrum as composed of two strong peaks at 228.90 and 232.02 eV, corresponding to Mo^4+^ 3d_5/2_ and Mo^4+^ 3d_3/2_, respectively.^[^ ^28^ ^]^ Figure 1h shows that the S 2p spectrum is composed of two peaks at 161.70 and 162.89 eV, corresponding to S 2p_3/2_ and S 2p_1/2_, respectively. These spectra are consistent with the reported values for MoS_2_ single crystal, indicating that the as‐exfoliated MoS_2_ nanosheets are of pure 2H phase and no oxidation is observed.^[^ ^27^ ^]^

Transmission electron microscopy (TEM) was used to investigate the atomic structure of the MoS_2_ nanosheets. Figure 1i shows the low‐magnification TEM image of a large MoS_2_ nanosheet exhibiting transparent morphology and a folded edge. High‐resolution TEM in Figure 1j reveals the highly crystalline structure of the exfoliated MoS_2_. Furthermore, the high angular dark field (HAADF) image in Figure 1k shows the defect‐free honeycomb lattice arrangement of the MoS_2_ crystal with typical (100) interplanar spacing of 0.28 nm. This result is consistent with the corresponding fast Fourier transform (FFT) pattern (Figure 1l), confirming the hexagonal symmetry of the atomic arrangement in semiconducting MoS_2_ and that the nanosheet consists of a single crystal domain,^[^ ^23^, ^36^ ^]^ hence validating the high crystalline quality of the as‐produced MoS_2_ nanosheets.

Inkjet printable and water‐based graphene and h‐BN inks were prepared by liquid‐phase exfoliation assisted with pyrene derivatives, following the approach already reported in literature (see Experimental sections for details).^[^ ^37^, ^38^ ^]^ The h‐BN ink is selected due to its good dielectric properties and minimal leakage current, as well as its compatibility with channel materials.^[^ ^39^, ^40^ ^]^

### 2.2 Channel Fabrication and Characterization

**Figure 2:**
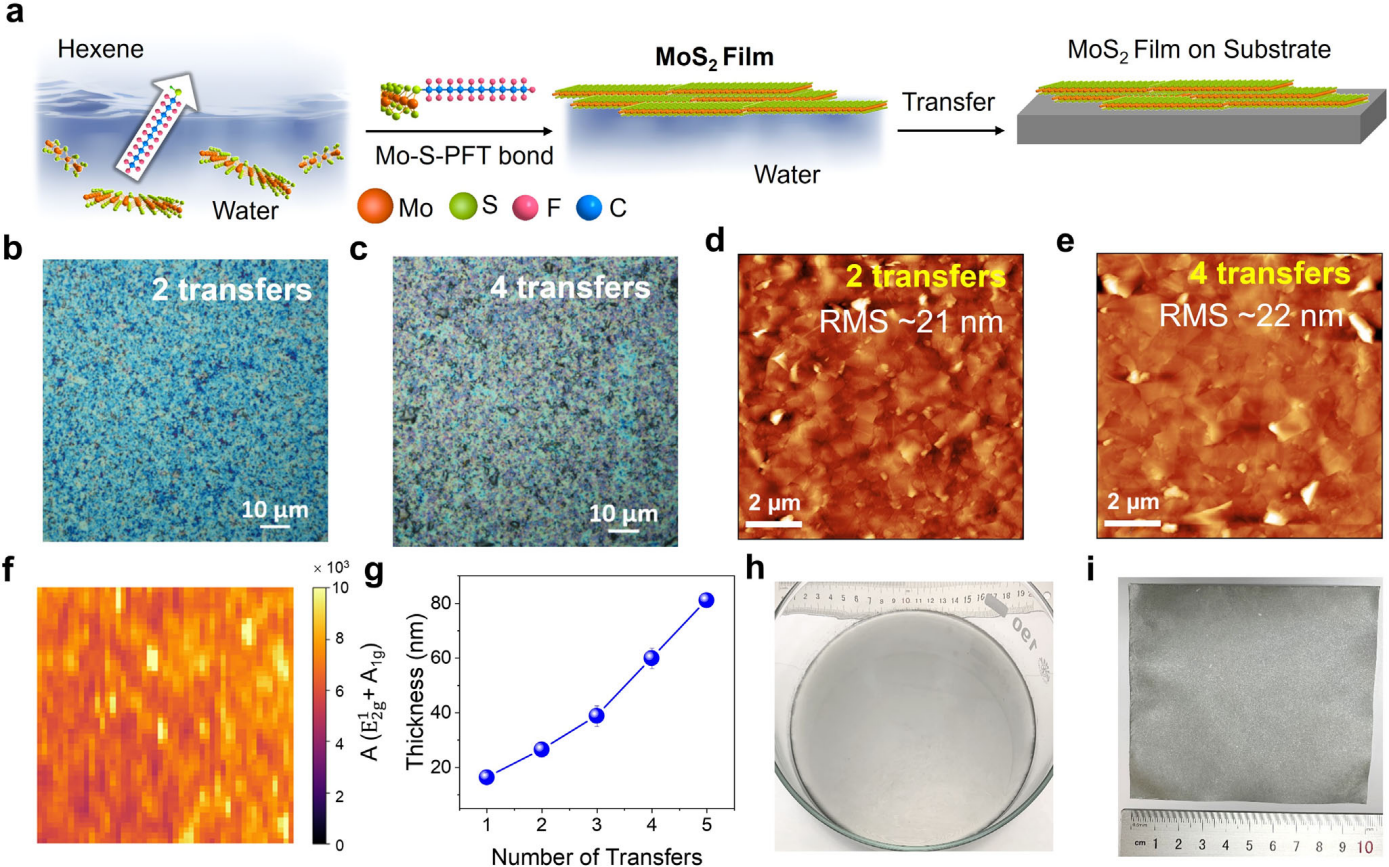
a) Schematic of the MoS_2_ film self‐assembly process. Optical pictures of MoS_2_ films obtained with b) 2 transfers and c) 4 transfers, respectively. (c) AFM images of MoS_2_ films obtained with d) 2 transfers and e) 4 transfers, respectively. f) Thickness of the films obtained by the AFM measurements vs the number of transfers. g) Raman map (150 × 150 µm^2^) of the integrated intensity of the $E_{2g}^{1}$ and A_1g_ peaks of a MoS_2_ film obtained with 2 transfers, showing full coverage. h) MoS_2_ film (diameter 19 cm) on the water surface and i) large area film transferred onto 10 × 10 cm^2^ size paper.

LLI assembly is used to fabricate MoS_2_ thin films, which are then transferred onto rigid and flexible substrates. The as‐produced MoS_2_ nanosheets (see Experimental sections for details) are first dispersed in DI water with the addition of 1‐pyrenesulfonic acid sodium salt (PS1), used as stabilizer, ^[^ ^41^ ^]^ via ultrasonication for 2 h. The dispersion is then mixed with an ethanol:hexane (mixing ratio 1:4) solution containing 0.1 m PFT, which facilitates the spontaneous assembly of the MoS_2_ nanosheets at the water‐hexane interface through the Mo‐S‐PFT bond that drives the assembly at the interface, **Figure** **2**a. The self‐assembled MoS_2_ thin films formed at the liquid‐liquid interface can be easily transferred onto both rigid and flexible substrates, while the film thickness can be controlled by repeating the transfer process several times. Figure 2b,c shows the optical microscope images of the MoS_2_ films deposited onto a Si/SiO_2_ substrate, made by repeating the transfer process 2 and 4 times, respectively. The AFM measurements of the MoS_2_ films obtained with 2 and 4 transfers show uniform coverage and an average RMS roughness of ≈21 nm in both cases (Figure 2d,e). Figure 2f shows a Raman map of the intensity sum of the $E_{2g}^{1}$ and A_1g_ peaks, measured on the MoS_2_ film obtained with 2 transfers, taken on an area of 150 µm × 150 µm, showing full coverage of the silicon substrate. Figure 2g shows that the thickness of each transferred film is ≈15 nm.

The PFT‐driven ILL approach offers significant potential for scale‐up fabrication, since the lateral size of the film is only limited by the size of the container and the substrates onto which the film is transferred. As a demonstration, Figure 2h,i shows a MoS_2_ film assembled on the water surface within a 19 cm diameter glass beaker, and a 10 × 10 cm^2^ MoS_2_ film transferred on paper, respectively.

### 2.3 Device Fabrication and Characterization

**Figure 3:**
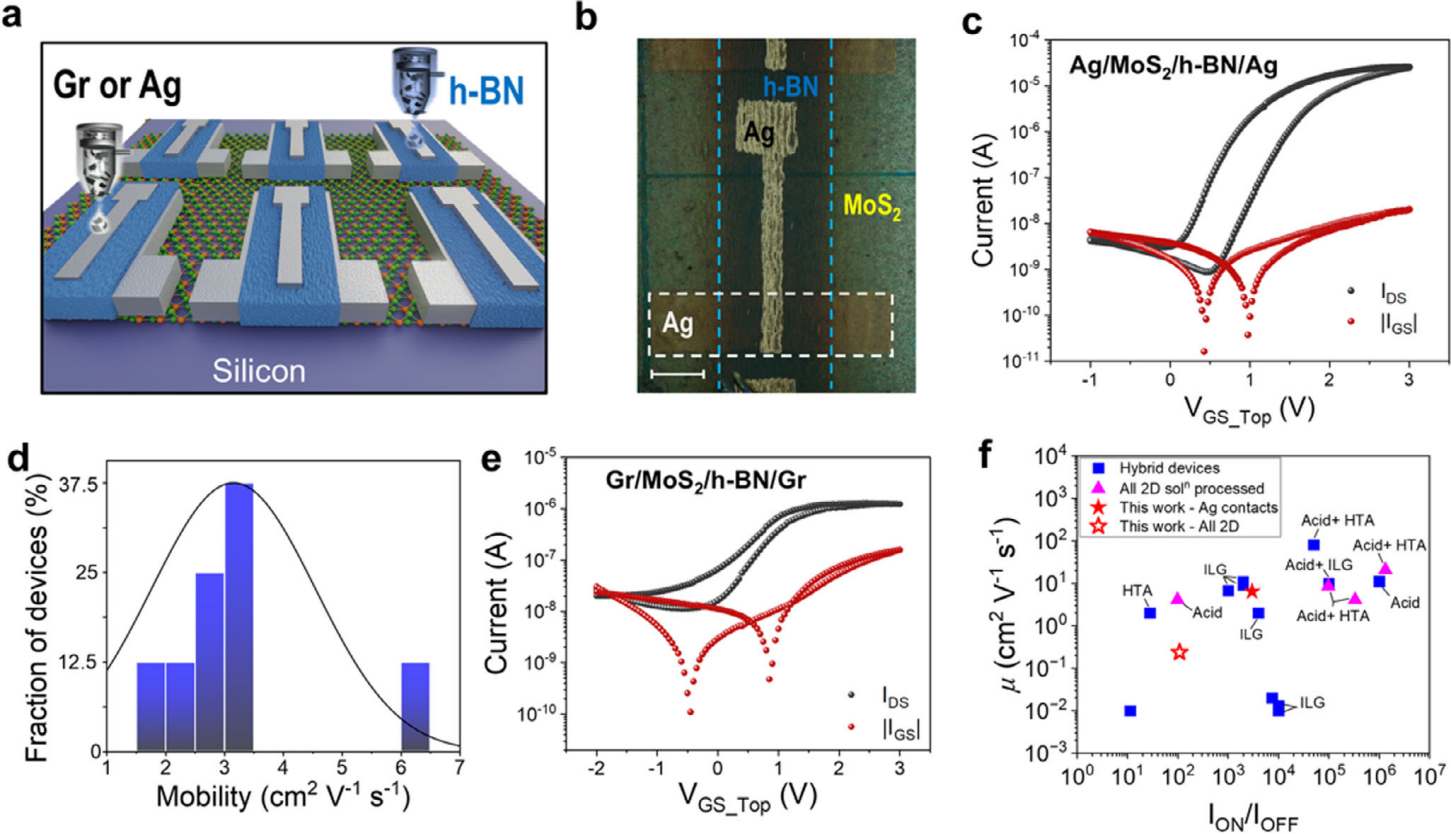
a) Schematic representation of the transistor with silver or graphene (Gr) contacts, h‐BN as dielectric, and MoS_2_ as semiconductor onto the Si/SiO_2_ substrate. b) Optical image of the transistor with silver contacts on a SiO_2_ substrate. Scale bar = 200 µm. c) Typical transfer characteristics of a MoS_2_ transistor with Ag contacts and h‐BN dielectric, measured as a function of the top‐gate voltage for a drain voltage (V_DS_) of 1.25 V. Logarithmic scale: black dots, drain current; red dots, gate current. d) Statistical distribution of charge carrier mobility obtained from 8 individual MoS_2_ transistors with printed Ag contacts and h‐BN dielectric. e) Typical transfer characteristic curve of a MoS_2_ transistor with Gr contacts and h‐BN dielectric, measured as a function of the top‐gate voltage for a drain voltage of 0.75 V. Logarithmic scale: black dots, drain current; red dots, gate current. f) State‐of‐the‐art comparison of field‐effect mobility (*µ*) and *I*_*ON*_/*I*_*OFF*_ of this work with previously reported TMDs‐based TFTs on rigid substrates.^[^ ^6^, ^7^, ^8^, ^9^, ^10^, ^12^, ^16^, ^42^, ^43^, ^44^, ^45^, ^46^ ^]^ Hybrid devices are defined when solution‐processed TMD films are combined with either solution‐processed conducting or insulating thin films, or when ILG is used. Labels indicate post‐processing treatments used in previous studies – Acid: TFSI‐treated channel; HTA: high temperature annealing (>300 °C); ILG: ionic‐liquid gating. Reference details are provided in Table S5 (Supporting Information).

The TFT channel was fabricated using the ≈60 nm thickness films achieved with 4 transfer (Figure 2g) and transferred onto a silicon substrate. This thickness was selected for the fabrication of the TFTs because at least 3 transfers are needed to produce a pin‐hole film, i.e. a film with thickness above its RMS (Figure 2d,g). The source and drain contacts are first printed with commercial silver ink onto the MoS_2_ film to define the channel area of the transistor (length 60 µm; width 600 µm). Finally, the h‐BN ink is printed onto the MoS_2_ channel, and the top‐gate electrode is printed with the silver ink onto the h‐BN film (**Figure** **3**b). The silver ink achieves high conductivity with just one printing pass, and it has shown ohmic contact with MoS_2_.^[^ ^39^ ^]^ Figure 3c shows the typical transfer characteristics of the TFT in Top‐Gate (TG) geometry. The devices work in the enhancement mode, and can operate at low supply voltage (<3 V), and have a threshold voltage (*V_TH_*) in the range of 1.5 V. the leakage current *I_GS_* (red dots, Figure 3c) through the insulator is negligible when the device is in the ON state, as compared to the drain current *I_DS_* (black dots, Figure 3c), further confirming the good insulating properties of the inkjet‐printed hBN film. The devices fabricated show an average on/off ratio (*I_ON_*/*I_OFF_*) of 1.5 × 10^3^ with an average subthreshold slope of 267 mV dec^−1^ (obtained over 8 different devices, as shown in Figure S9 and Table S1, Supporting Information). To evaluate the effective carrier mobility, the device was fabricated in the back‐gate (BG) configuration using the SiO_2_ (290 nm thickness) of the silicon substrate as the gate dielectric (Figure S10a, Supporting Information), with the same geometric parameters used for the TG device in Figure 3b, and by printing the drain and source electrodes on top of the MoS_2_ with the silver inks. The extracted charge carrier mobility of the device, operating in the linear regime ($V_{DS_{BG}}$ = 3V), as illustrated in Figure S10c (Supporting Information), is 0.4 cm^2^ V^−1^ s^−1^. Using the results obtained with this BG configuration, the average capacitance value of the printed h‐BN film, *C_hBN_*, is 760 nF cm^−^^2^ (details in Section S3 of Supporting Information). Using the calculated capacitance of printed h‐BN gate dielectric, the average mobility of the FETs with Ag contacts is 3.16 ± 1.39 cm^2^ V^−1^ s^−1^ (with best performance reaching up to 6.29 cm^2^ V^−1^ s^−1^), Figure 3d.

The all‐2D material‐based TFT can be fabricated by replacing the silver ink with graphene for printing the source, drain, and top contacts (Figure S11a, Supporting Information), using the same configuration shown in Figure 3b, hence demonstrating a fully solution‐processed TFT made of 2D materials. The transfer characteristics are shown in Figure 3e. The higher gate current, as compared to the device made with silver, can be attributed to the higher porosity and roughness of the graphene printed film, as compared to the silver film, thus increasing the risk of parasitic conduction pathways between the gate and the source/drain electrodes. The *I_DS_* value is also lower as compared to the same device made with silver contacts because of the higher resistance of the graphene contacts as compared to silver contacts. The average mobility value found for devices on a rigid substrate with graphene contacts, using the previously estimated capacitance value *C_hBN_*, is 0.19 cm^2^ V^−1^ s^−1^ (with best performance reaching up to 0.23 cm^2^ V^−1^ s^−1^). The transfer characteristics of all 3 TFTs are shown in Figure S11b (Supporting Information). Table S2 (Supporting Information) summarizes the key device performance parameters.

Figure 3f compares our all‐2D material TFTs made on silicon with state‐of‐the‐art TMDs‐based TFT, either fully solution processed or hybrids (i.e., with at least two components made by solution‐processed thin films). Note that in this framework, the IL is not a solid thin film, so all devices using ILG are considered “hybrids” (see Table S3, Supporting Information for details). Our devices with silver contacts show comparable performance to devices with IL gating, made without acid treatment.^[^ ^7^, ^16^ ^]^ To note that I_ON_/I_OFF_ of our devices with silver contacts is below that of all solution‐processed TFTs on silicon; however, these devices use oxidized HfS_2_, hence requiring annealing at 500 °C.^[^ ^6^, ^9^, ^10^ ^]^ While this approach can be used for glass or silicon substrates, it is unsuitable for flexible substrates. Finally, Figure 3f shows that our devices with graphene contacts have comparable performance reported for hybrid devices fabricated on Si/SiO_2_ substrate, without acid treatment or ILG, which typically exhibit mobilities below 1.0 cm^2^ V^−1^ s^−1^ and *I_ON_*/*I_OFF_* ratios of ≈10^2^.^[^ ^6^, ^20^, ^47^ ^]^ However, it is worth noting that all our device measurements were conducted under ambient conditions, which can significantly affect the device performance due to the chemisorption of oxygen and water molecules on MoS_2_, which act as charge traps and scattering centres,^[^ ^48^, ^49^, ^50^, ^51^ ^]^ when the device is measured in BG. Hence, the h‐BN dielectric layer is expected to better protect the MoS_2_ channel, enabling stable performance under ambient conditions.

**Figure 4:**
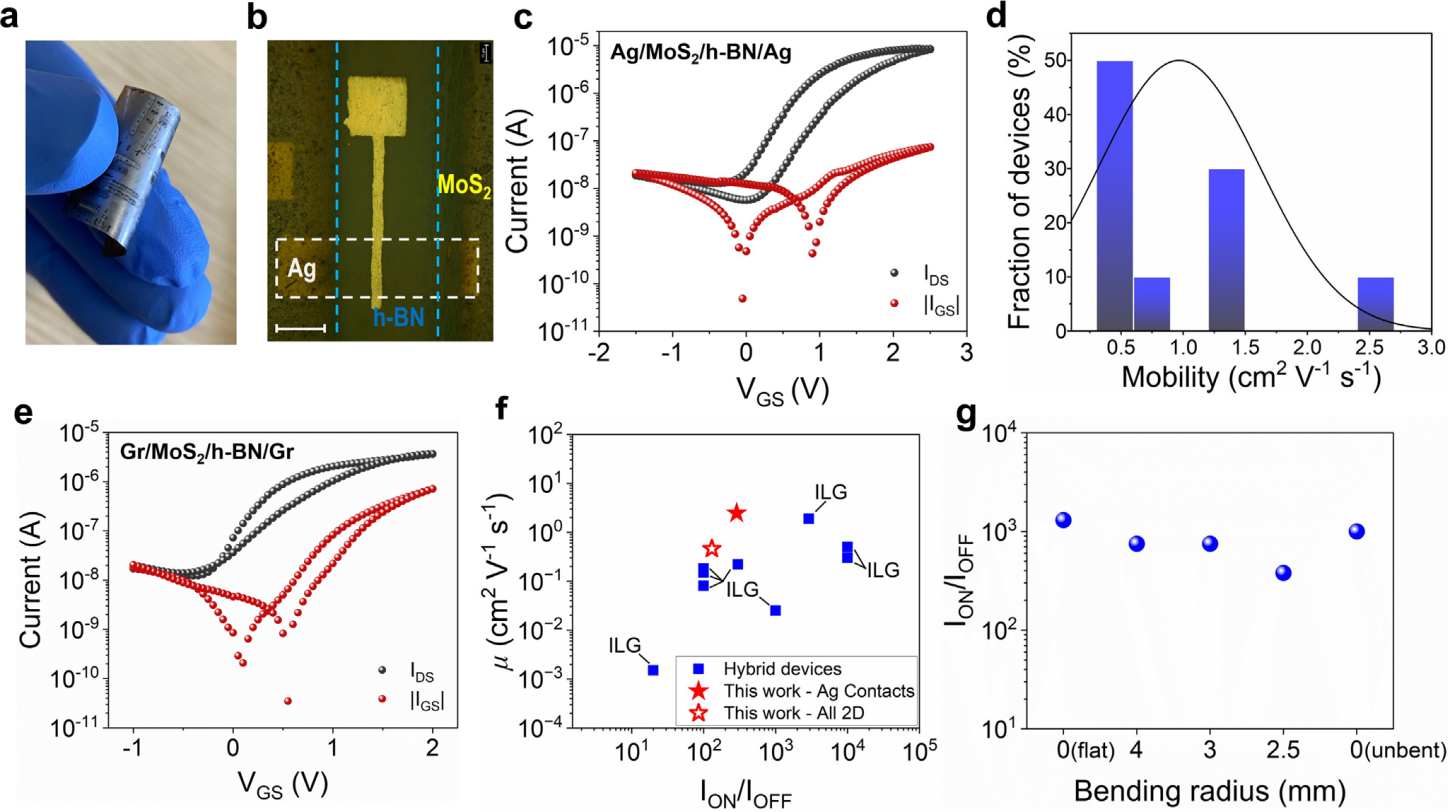
a) Photograph of a flexible MoS_2_ TFT made on a polyimide substrate. b) Optical image of the transistor with silver contacts onto the PI substrate. Scale bar = 200 µm. (c) c) Typical transfer characteristics of a MoS_2_ transistor with Ag contacts and h‐BN dielectric film, measured as a function of the top‐gate voltage for a drain voltage (V_DS_) of 1.25 V. Logarithmic scale: black dots, drain current; red dots, gate current. d) The statistical distribution of mobility obtained from 10 individual MoS_2_ transistors on PI substrate with printed Ag contacts and h‐BN dielectric film. e) Typical transfer characteristic curve of a MoS_2_ transistor with Gr contacts and h‐BN dielectric film, measured as a function of the top‐gate voltage for a drain voltage of 1.25 V. Logarithmic scale: black dots, drain current; red dots, gate current. f) State‐of‐the‐art comparison of field‐effect mobility (*µ*) and *I*_*ON*_/*I*_*OFF*_ from this work with previously reported solution‐processed TMDs‐based TFTs onto flexible substrates.^[^ ^7^, ^8^, ^52^, ^53^, ^54^, ^55^ ^]^ Hybrid devices are defined when solution‐processed TMD films are combined with either solution‐processed conducting or insulating thin films, or when ILG is used. Labels indicate post‐processing treatments used in previous studies – Acid: TFSI treated channel; HTA: high temperature annealing (>300 °C); ILG: ionic‐liquid gating. Reference details are provided in Table S5 (Supporting Information). g) *I*_*ON*_/*I*_*OFF*_ ratio of MoS_2_ TFTs onto PI under different bending radius and back to flat condition (unbent).

Our approach is successfully used for the fabrication of MoS_2_‐based TFT (device schematic shown in Figure S12, Supporting Information) onto polyimide (PI) substrate, enabling flexible TFTs (**Figure** **4**a). Figure 4b shows the optical microscope image of one of the devices. Figure 4c,f show the typical transfer characteristics of the TFT fabricated onto PI using silver and graphene, respectively, as contacts. The devices fabricated with silver contacts show an average *I_ON_*/*I_OFF_* ratio of 2.02 × 10^2^, an average subthreshold slope of 420 mV dec^−1^ and an average mobility of 0.98 ± 0.57 cm^2^ V^−1^ s^−1^,(with best performance reaching up to 2.47 cm^2^ V^−1^ s^−1^), Figure 4d. The devices can operate at low supply voltage (<3 V) and exhibit a threshold voltage (*V_TH_*) in the range of 1.4 V. Figure S13 (Supporting Information) shows the transfer characteristics of 10 different devices onto PI, and Table S3 (Supporting Information) summarizes the key performance parameters of all devices tested.

The transfer characteristics of the devices fabricated with graphene contacts (optical microscopy image of the device shown in Figure S14a, Supporting Information) are shown in Figure 4e. The devices show an average *I_ON_*/*I_OFF_* ratio of 1.3 **×** 10^2^ and an average subthreshold slope of 620 mV dec^−1^, measured on 4 devices (Figure S14b, Supporting Information). The devices can operate at low supply voltage (<3 V) and exhibit *V_TH_* in the range of 0.4 V (Figure S14b, Supporting Information). The use of graphene contacts shows the very same limitations as those encountered in their rigid counterparts. Specifically, we observe large gate leakage currents accompanied by diminished drain currents, indicating sub‐optimal charge transport characteristics at the graphene‐MoS_2_ interface, although the decrease in performance is less pronounced in comparison to the devices made on silicon. (Figure 3f). The average *µ*_FE_ on flexible substrate with graphene contacts is 0.23 ± 0.14 cm^2^ V^−1^ s^−1^ (with best performance reaching up to 0.46 cm^2^ V^−1^ s^−1^). Table S4 (Supporting Information) summarizes the key performance parameters of all devices tested.

Figure 4f benchmarks the performance of our TFT devices against previously reported TMD‐based FETs onto flexible substrates (Table S5, Supporting Information for details).^[^ ^7^, ^8^, ^52^, ^53^, ^54^, ^55^ ^]^ To the best of our knowledge, this is the first demonstration of an all‐2D TFT onto a flexible substrate without ILG, demonstrating comparable performance and stable operation in ambient conditions.

Finally, to fully demonstrate the compatibility of our device for flexible electronics, the electrical characteristics were measured under bending conditions (Figure S15, Supporting Information). Figure 4g shows the changes in the *I_ON_*/*I_OFF_* of the device up to a radius of curvature (*R*) of 2.5 mm and back to the flat condition (*R* = 0), showing stable device performance.

## 3 Conclusion

This work demonstrates a fully solution‐processed strategy for the fabrication of all 2D material‐based TFTs on both rigid and flexible substrates without using ionic‐liquid gating, acid treatment, and high temperature (> 300 °C) processing. By combining inkjet printing for fabrication of the graphene (or silver) electrodes and the h‐BN dielectric film, with a supramolecular liquid‐liquid interface assembly for fabrication of the semiconducting MoS^2^ film onto rigid and flexible substrates, we achieved all 2D‐material TFTs made onto flexible substrates with low‐voltage operation (<3 V), negligible leakage current, and mobilities up to 2.47 and 0.46 cm^2^ V^−1^ s^−1^ with silver and graphene electrodes, respectively. Furthermore, the devices are stable in air and under a bending radius of at least 2.5 mm.

The supramolecular assembly approach enables rapid deposition of highly crystalline, monolayer‐rich MoS_2_ films with excellent uniformity, by overcoming the limitations of traditional liquid‐liquid assembly techniques, while the use of h‐BN enables to fabrication of the device using processing temperature below 500 °C, in contrast to metal oxide films.

In conclusion, this work establishes a simple and scalable platform for next‐generation flexible electronics. Validation of this approach for the fabrication of arrays or more complex circuits will help position solution‐processed 2D materials at the forefront of future device technologies.

## 4 Experimental Section

### Electrochemical Exfoliation of 2D Layered Crystals

Cathodic intercalation of layered 2D crystals was carried out in a two‐electrode electrochemical cell. A thin piece of MoS_2_ crystal (Manchester Nanomaterials Ltd.) was fixed on a copper tape as a cathode, and a platinum foil (Sigma–Aldrich) served as an anode, respectively. Both electrodes were immersed in a solution of tetrapropylammonium chloride (TPA^+^Cl^−^) (purity 98%, Sigma–Aldrich) dissolved in propylene carbonate (PC) and used as an electrolyte. Three different concentrations of the TPA^+^Cl^−^ (i.e., 5 mm, 1 mm, and 0.5 mm) were used to optimize the exfoliation processes. To exfoliate MoS_2_ crystals, a voltage of ‐10 V vs the Pt was applied on the cathode for 1 h. Subsequently, the delaminated flakes from the electrode were subjected to bath sonication for 10 min for further exfoliation. Finally, the exfoliated MoS_2_ dispersion was centrifuged with a 1–14 k refrigerated centrifuge at 1500 rpm for 5 min to sediment the un‐exfoliated materials and extract the supernatant containing thin MoS_2_ nanosheets.

### Preparation of Inkjet Printable 2D Materials Inks

Inkjet printable and water‐based graphene and h‐BN inks were prepared by the liquid‐phase exfoliation, as described in previous works.^[^ ^37^, ^38^ ^]^ Briefly, bulk graphite (Sigma–Aldrich, 99.5% grade) and bulk h‐BN (Sigma–Aldrich, >1 µm, 98% grade) powders were used to prepare the inks. The bulk powders were dispersed in de‐ionized water at a concentration of 3 mg mL^−1^ and PS1, purchased from Sigma–Aldrich (purity ≥97%), was added at a concentration of 1 mg mL^−1^. The DI water was first degassed at 100 °C for 15 min and cooled down to room temperature under nitrogen. The graphite and h‐BN dispersions were sonicated for 120 h using a 300 W Hilsonic HS 1900/Hilsonic FMG 600 bath sonicator at 20 °C. The resultant dispersions were centrifuged at 3500 rpm (903g) for 20 min using a Sigma 1–14K refrigerated centrifuge to separate out and discard the residual bulk and non‐exfoliated flakes. The remaining supernatant was centrifuged twice at 16000 rpm (16602g) for 60 min to remove excess PS1 from the dispersion. After washing, the precipitate was redispersed in the printing solvent, as described in ref. [^[^ ^37^ ^]^] The concentration of the prepared inks was assessed using a Varian Cary 5000 UV–Vis spectrometer and the Beer‐Lambert law, using extinction coefficients of 2460 L g^−1^ m^−1^ (at 660 nm) and 1000 L g^−1^ m^−1^ (at 550 nm) for graphene^[^ ^56^ ^]^ and h‐BN,^[^ ^57^ ^]^ respectively. The ink concentration was set at ≈2 mg mL^−1^ to avoid nozzle blockage. The average lateral sizes of the nanosheets were 174 and 160 nm for graphene and h‐BN, respectively, with corresponding average thicknesses were 6.8 and 6.5 nm, respectively.^[^ ^58^, ^59^ ^]^ The nanosheets have high crystallinity and no oxidation.^[^ ^58^, ^59^ ^]^

### Interfacial Assembly of MoS_2_

The as‐prepared ECE MoS_2_ dispersion in PC was first centrifuged at 15000 rpm (16602 g) for 1 h. The sedimented material was re‐dispersed in deionized (DI) water containing PS1 (1 mg mL^−1^), followed by ultrasonication at 600 W using a Hilsonic bath sonicator for 2 h. Subsequently, the MoS_2_ dispersion in water was centrifuged at 1500 rpm for 10 min using a Sigma 1–14 k refrigerated centrifuge to remove aggregated flakes. The remaining supernatant, containing thin MoS_2_ flakes, was subjected to two additional centrifugation steps at 15000 rpm (16602 g) for 1 h each to eliminate excess PS1 from the dispersion.

To fabricate thin films by interfacial assembly, the aqueous solution of PS1‐stabilized ECE MoS_2_ (10 mL, concentration 0.1 mg mL^−1^) was placed into a 50‐mL glass bottle. Then, the PFT (Sigma–Aldrich, purity 97%) solution (0.1 mM, 10 mL in the ethanol and hexane mixed solvent with V_ethanol_:V_hexane_ = 1:4) was poured into the MoS_2_ solution, by oscillating for 3 s and allowing it to stand for 2 s till obtaining a dense and uniform MoS_2_ thin film at the hexane‐water interface. After a uniform film was formed, the hexane was allowed to evaporate, and subsequently, the transfer to the substrate was performed.

### Transfer of the MoS_2_ Film

The MoS_2_ film was transferred from the air‐water interface onto the target substrate, such as a SiO_2_/Si, PI, and paper, using scooping. Prior to the film transfer process, both the SiO_2_/Si and PI substrates were cleaned by bath sonication in acetone and 2‐propanol for 15 min in each solvent and subsequently dried under a stream of N_2_. The clean and dry substrate (e.g., silicon wafer) was slowly and vertically placed into the water with the surface intended for the film transfer facing down. The substrate should be carefully aligned and inserted at a constant speed to avoid disturbing the film on the water surface. After full contact of the film with the substrate, the film was slowly lifted out from the water at a constant speed to prevent breakage as well as ensure uniform transfer. After the transfer, the thin film on the substrate was left to dry under ambient conditions, and then it was gently rinsed with DI water to clean the surface, and then dried using a gentle flow of nitrogen gas or at room temperature. The transfer can be repeated by depositing several transferred films one on top of the other. The final film, obtained with one of multiple transfers, was then annealed at 300 °C for 2 h under vacuum to remove residual solvent.

### Devices Fabrication

MoS_2_ thin film transistors were fabricated in both top‐gate and back gate configuration on the MoS_2_ transferred film on Si/SiO_2_ substrate (Inseto UK, n‐doped Si with resistivity of ≈10 Ω·cm, corresponding to a phosphorous dopant density of ≈4.5 × 10^14^ cm^−3^, covered with 290 nm thermally grown SiO_2_ and in top gate configuration on PI (BENECREAT Thickness 50 µm). A Dimatix Materials Printer 2850 (Fujifilm) was used to define the contacts and the insulator layers under ambient conditions. Cartridges with a droplet volume of 2.4 pL were used for the definition of both silver and graphene contacts.

The silver ink was deposited with a single printing pass using one nozzle, a drop spacing of 30 µm, and by keeping the printer platen at room temperature. It was worth underlining that no annealing or post‐treatment process was performed after any printing step when silver was used. When the graphene ink was employed, source and drain contacts were inkjet‐printed using a drop spacing of 20 µm and 30 printing passes. Annealing at 150 °C for 1h was performed after contact fabrication to remove residual water. For the top‐gate contacts, only 15 printing passes of graphene ink were used in order to reduce the possibility of overlapping with the source and drain contacts at each print pass. The h‐BN ink was printed on top of the MoS_2_ film using a drop spacing of 20 µm and 150 printing passes. Several thin‐film transistors have been fabricated on both rigid and flexible substrates and characterized with a nominal width‐length ratio of 10 (further details can be found in Tables S1 and S3, Supporting Information).

### Characterisation

A Varian Cary 5000 UV–Vis spectrometer was used to measure the absorption spectrum of the MoS_2_ dispersion. The samples for optical microscopy, AFM, Photoluminescence (PL), and Raman characterisations were prepared by drop casting the supernatant on pre‐cleaned SiO_2_/Si substrates, followed by annealing at 300 °C for 1 h under vacuum. Optical microscopy images were taken with a Nikon Eclipse LV100 microscope. The lateral flake sizes of the MoS_2_ nanosheets were calculated by analysing the images with ImageJ software. AFM measurements were taken with a Bruker Multimode 8 Atomic Force Microscope operating in PeakForce Tapping mode, with ScanAsyst‐Air cantilevers employed for all measurements. Both the Raman and PL measurements were performed using a Renishaw InVia Raman spectrometer equipped with a 514.5 nm excitation line using a 100X objective lens and using 0.3 mW laser power.

The X‐ray photoelectron spectroscopy measurements were performed using the K‐Alpha X‐ray Photoelectron Spectrometer System from Thermo Scientific. The photon source was a monochromatized Al K α line (hν = 1486.6 eV). The spectra were acquired using a spot size of 300 µm and constant pass energy (150 eV for survey and 20 eV for high resolution spectra). A flood gun with combined electrons and low‐energy Ar ions was used during the measurements. HRTEM images were acquired on a JEOL 2100‐F microscope with a field‐emission gun operated at 200 kV accelerating voltage, providing direct images of the atomic structure. A High‐Angle Annular Dark Field detector and an Oxford high solid‐angle Silicon Drift Detector X‐Ray Energy Dispersive Spectrometer system were used for chemical elemental analysis.

The transistor characterization was carried out using a Keithley SCS4200 parameter analyzer. All the electrical measurements were performed in air under ambient conditions.

## Conflict of Interest

The authors declare no conflict of interest.

## Supporting information



Supporting Information

## Data Availability

The data that support the findings of this study are available from the corresponding author upon reasonable request.
