# Supplementary material for: Ionic‐Liquid Free and Flexible Transistors Made of 2D Material Inks
Source: Small. 2025 Oct 30;21(50):e08360. doi: 10.1002/smll.202508360 (PMC12710143; doi:10.1002/smll.202508360)
Supplement: Supplementary file 1 — Supporting Information [file SMLL-21-e08360-s001.pdf]

NANO · MICRO
small

Supporting Information

for *Small*, DOI 10.1002/smll.202508360

Ionic-Liquid Free and Flexible Transistors Made of 2D Material Inks

*Liming Chen, Khaled Parvez, Francesco Nepa, Elisabetta Dimaggio, Chaochao Dun, Oliver Read, Jeffrey J. Urban, Gianluca Fiori and Cinzia Casiraghi**

Supporting Information

Ionic-liquid Free and Flexible Transistors made of 2D Material Inks

*Liming Chen[1]†, Khaled Parvez[1]†, Francesco Nepa[2]†, Elisabetta Dimaggio[2], Chaochao Dun[3], Oliver Read[1], Jeffrey J. Urban[3], Gianluca Fiori[2], Cinzia Casiraghi[1]**

L. Chen, K. Parvez, O. Read, C. Casiraghi
Department of Chemistry,
University of Manchester,
Oxford Road, M13 9PL Manchester,
United Kingdom
E-mail: cinzia.casiraghi@manchester.ac.uk

F. Nepa, E. Dimaggio, G. Fiori
Dipartimento di Ingegneria dell'Informazione,
University of Pisa, Pisa,
Italy

C. Dun, J. J. Urban
Lawrence Berkeley National Laboratory,
Berkeley, CA 94720
USA

† Authors contributed equally

Content

Section 1. Ink production and characterization

Section 2. Electrical characterization on rigid substrate using top gate

Section 3. Electrical characterization on rigid substrate using back gate

Section 4: Calculating the capacitance of the inkjet printed hBN film

Section 5. Electrical characterization on flexible substrate

Section 6. Electrical measurements under bending

Section 1. Ink production and characterization

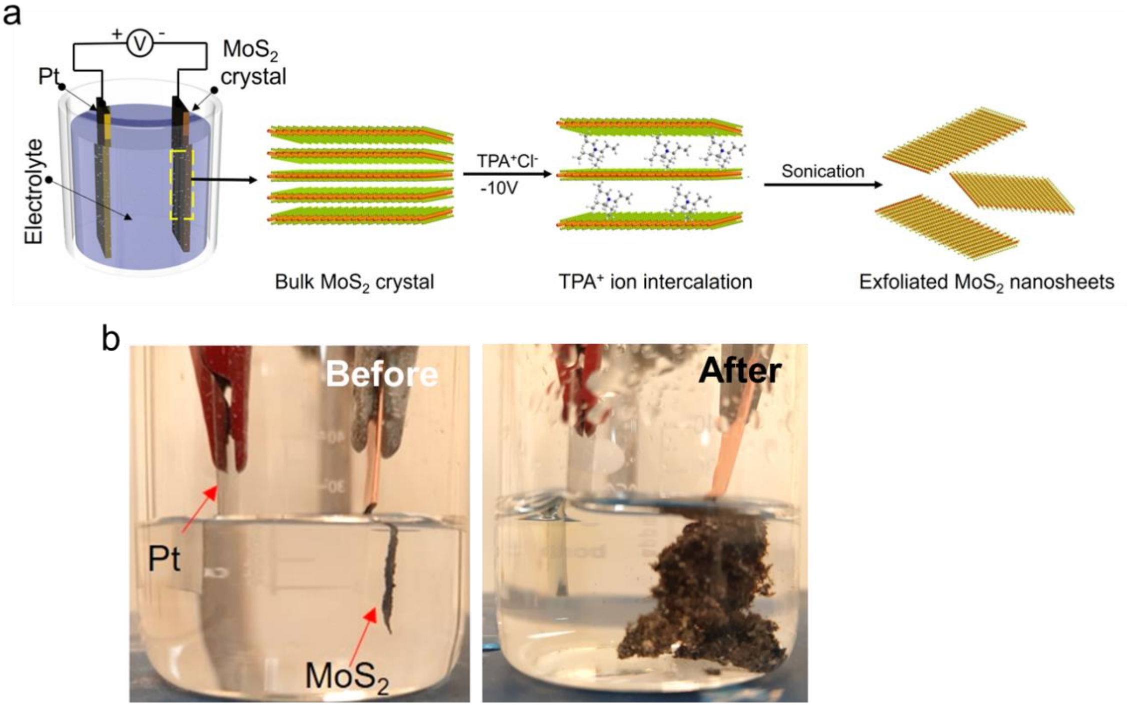

Figure S1: (a) Schematic illustration of the electrochemical exfoliation of MoS_2. (g) Photographs of the bulk MoS_2 crystal, before (left) and after exfoliation (right).

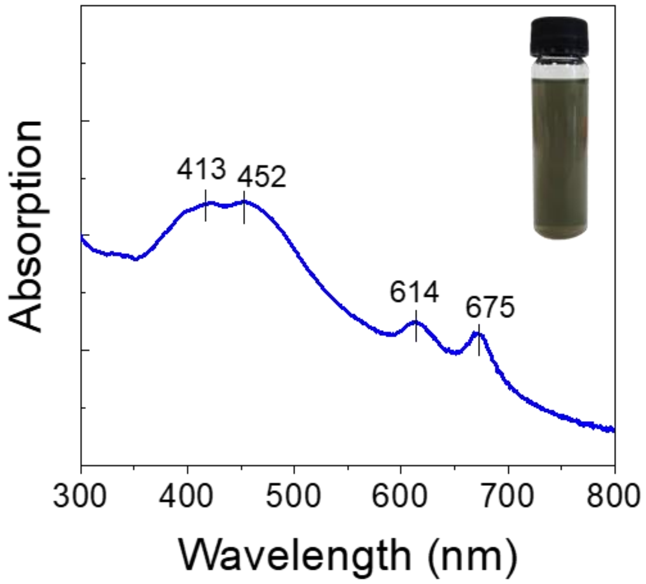

Figure S2: UV-Visible absorption spectrum of the exfoliated MoS_2 indicating four distinct absorption peaks. *Inset*: photograph of the MoS_2 dispersion in propylene carbonate (PC).

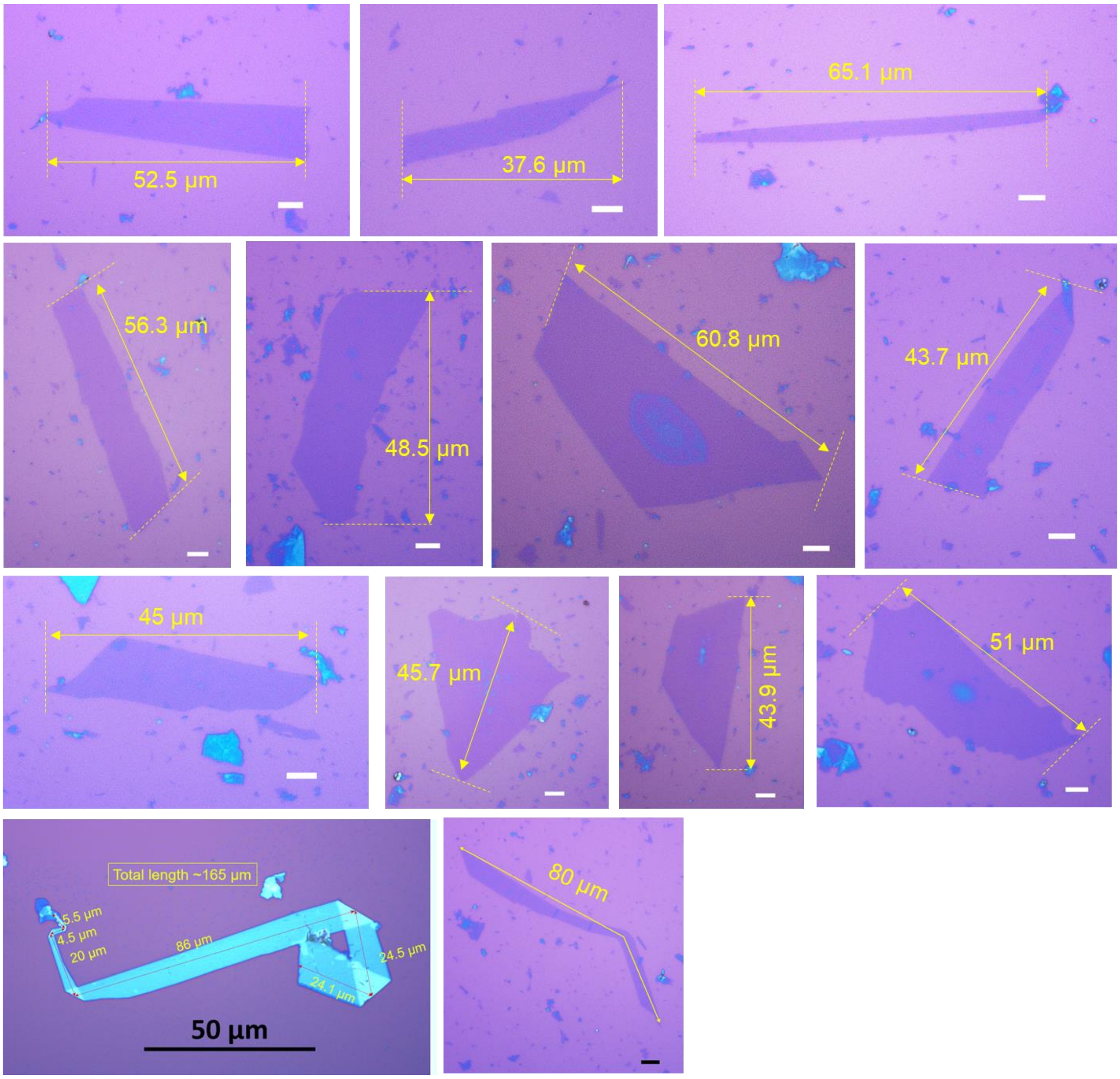

Figure S3: Optical microscopic images of large MoS_2 flakes produced by ECE. Scale bar: 5 µm.

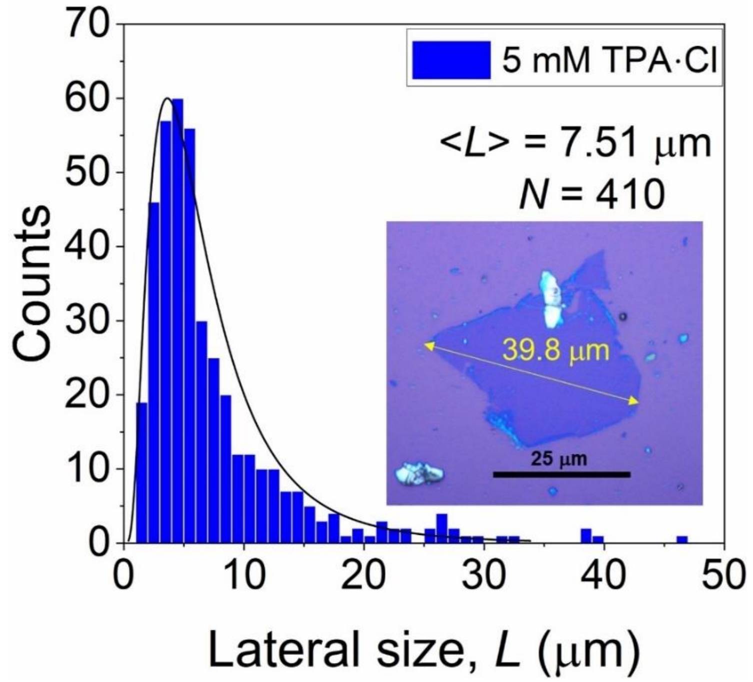

Figure S4: The lateral flake size distribution of ECE MoS_2 obtained at a concentration of 5.0 mM TPA^+Cl^-. *Inset*: optical microscopy image showing a representative MoS_2 nanosheet produced under this exfoliation condition.

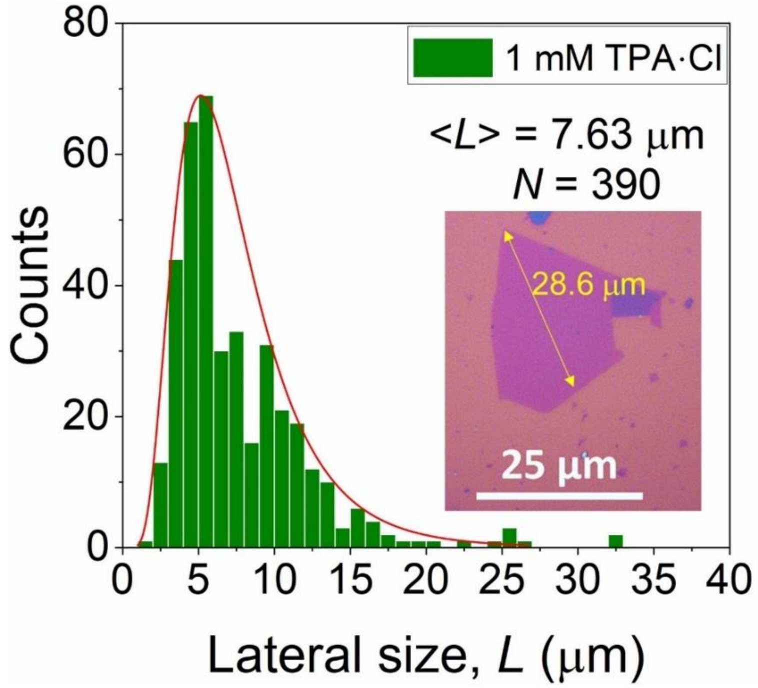

Figure S5: The lateral flake size distribution of ECE MoS_2 obtained at a concentration of 1.0 mM TPA^+Cl^-. *Inset*: optical microscopy image showing a representative ECE MoS_2 nanosheet produced under this exfoliation condition.

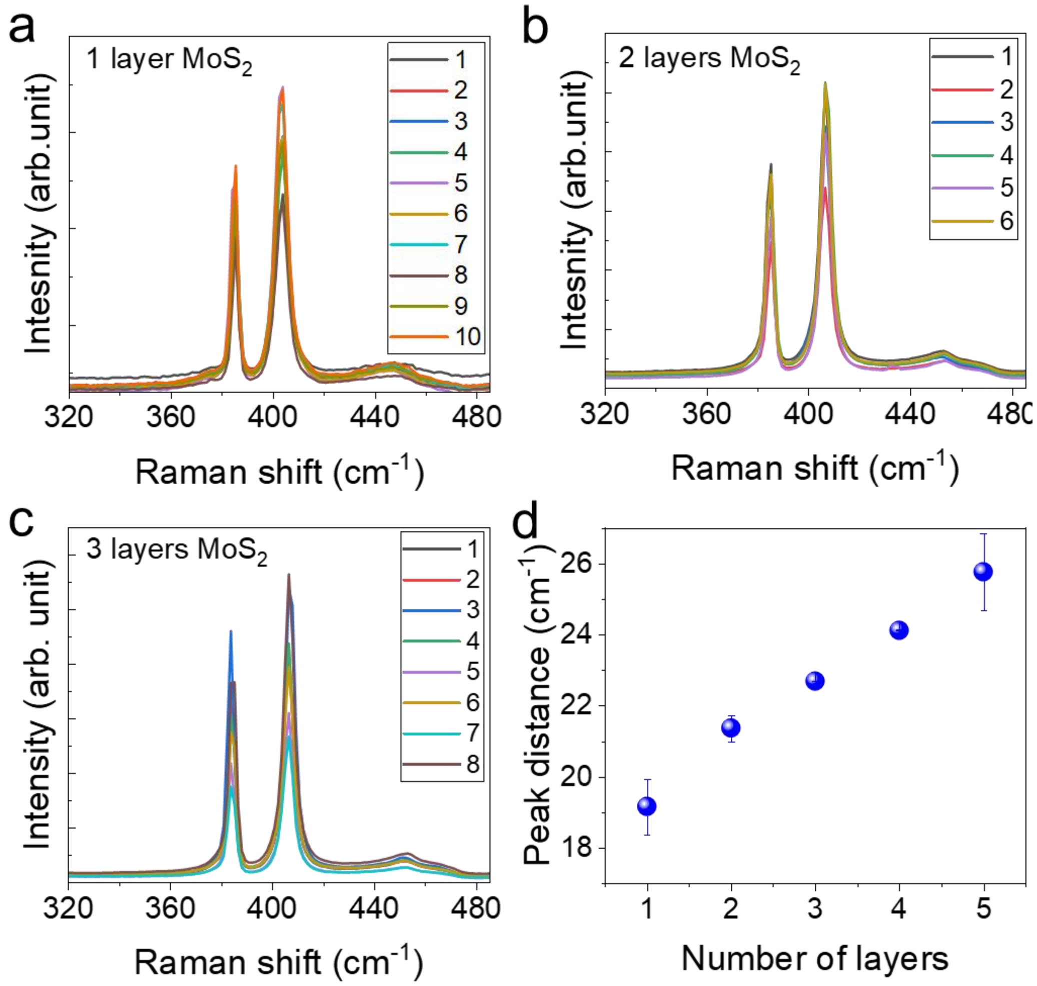

Figure S6: Representative Raman spectra of MoS_2 nanosheets made by ECE with: (a) monolayer, (b) bi-layer and (c) tri-layer thicknesses. (d) Raman peak separation between E^1_{2g} and A_{1g} vibrational modes as a function of the number of layers.

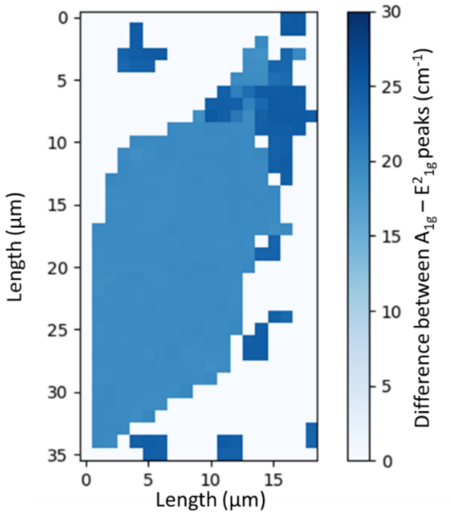

Figure S7: Raman map showing the difference between E^1_{2g} and A_{1g} peaks of a single layer MoS_2 nanosheet, highlighting the uniform thickness distribution.

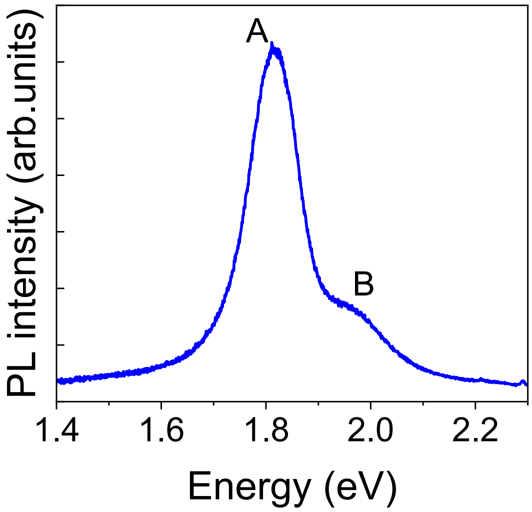

Figure S8: Photoluminescence spectra of a single layer electrochemically exfoliated MoS_2.

Section 2. Electrical characterization on rigid substrate using top gate.

Table S1 and Fig. S9, show the performance and the transfer characteristics of eight MoS_2 field-effect transistors fabricated with silver contacts on SiO_2 substrates. The eight MoS_2-based FETs exhibit typical n-type behaviour with V_{TH} around 1.5-2.0 V. The highest performing device demonstrates an on/off current ratio of 3.04×10^3 and a subthreshold slope of 325 mV dec^{-1}. Off-state currents are consistently low, ranging from 2.47 to 9.68 nA, indicating good gate control and minimal leakage. The transfer characteristics show similar switching behaviour across all devices, though with some variation in maximum on-current levels, suggesting good reproducibility in the fabrication process.

Table S1: Table summarizing key device parameters including off-current (I_{OFF}), on-current (I_{ON}), on/off ratio (I_{ON}/I_{OFF}), threshold voltage (V_{TH}), and subthreshold slope (SS).

	MoS_2 based FETs on SiO_2 substrate				
	I_{off} (μA)	I_{on} (μA)	I_{on}/I_{off}	V_{th} (V)	SS (mV/dec)
Device 1	9.68×10^{-9}	2.14×10^{-5}	2.21×10^{3}	1.62	145
Device 2	2.47×10^{-9}	7.00×10^{-6}	2.83×10^{3}	1.51	265
Device 3	2.55×10^{-9}	7.75×10^{-6}	3.04×10^{3}	1.53	325
Device 4	2.96×10^{-9}	8.13×10^{-7}	2.74×10^{2}	1.58	214
Device 5	3.72×10^{-9}	7.02×10^{-6}	1.89×10^{3}	1.63	220
Device 6	3.14×10^{-9}	7.59×10^{-7}	2.42×10^{2}	2.02	300
Device 7	4.65×10^{-9}	2.29×10^{-6}	4.92×10^{2}	1.94	295
Device 8	4.41×10^{-9}	2.21×10^{-6}	5.01×10^{2}	1.9	375

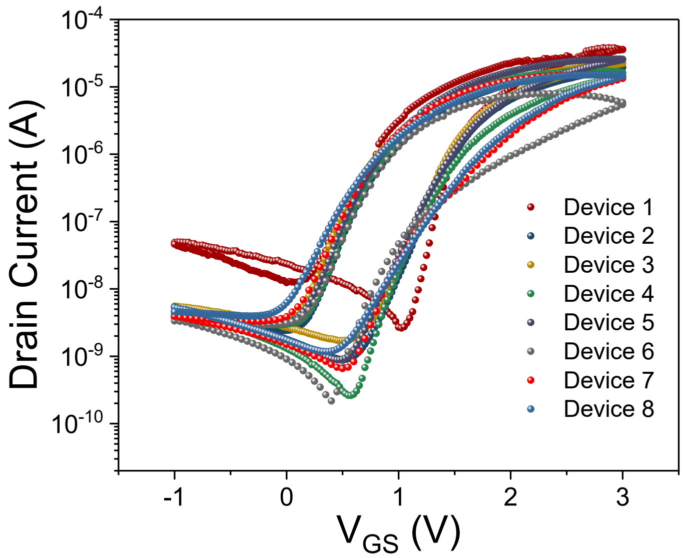

Figure S9: Transfer characteristics showing drain current (I_{DS}) vs. gate voltage (V_{GS}) curves for all devices, demonstrating consistent switching behaviour with slight variations in performance metrics. The devices exhibit typical n-type semiconductor behaviour with on/off ratios ranging from 2.42×10^2 to 3.04×10^3 and threshold voltages between 1.51 V and 2.02 V.

Section 3. Electrical characterization on rigid substrate using back gate.

The electrical performance of the field-effect transistors was evaluated using back-gate measurements on a rigid SiO_2 substrate (Figure S11a). The transfer curve in Figure 11b, plotted as drain current versus back-gate voltage, demonstrates n-type semiconductor behaviour. By analysing these measurements, we extracted the carrier mobility, which shows strong dependence on the applied gate voltage.

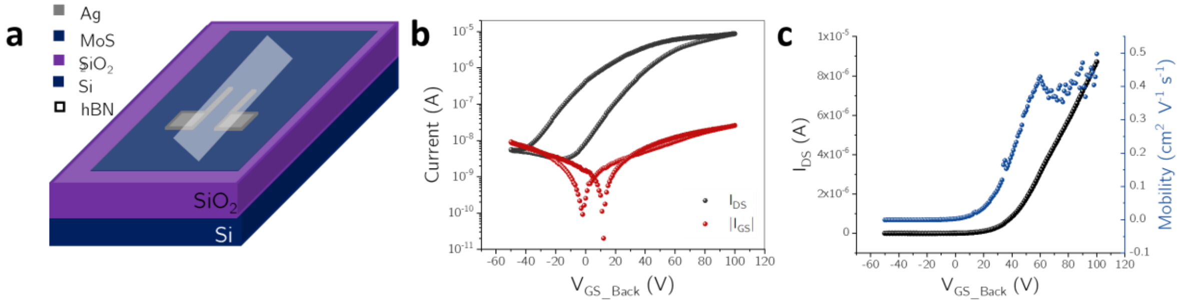

Figure S10: (a) Schematic representation of the transistor with silver contacts, SiO_2 as dielectric, and MoS_2 as semiconductor. (b) Typical transfer characteristic curve measured as a

function of the back-gate voltage for a drain voltage of 3V. Logarithmic scale: black dots, drain current; red dots, gate current. (c) Typical transfer characteristic curve measured as a function of the gate voltage for a drain voltage of 3 V in linear scale (black dots) and field-effect mobility (blue dots).

Section 4: Calculating the capacitance of the inkjet printed hBN film.

The relative capacitance value of the 150 passes inkjet-printed hBN, with the following procedure. First, the drain current for the back gate device in linear region, for low $V_{DS_{BG}}$ values, was expressed using Equation 1:

$$I_{DS} = \mu_{FE} C_{SiO_2} \frac{W}{L} \left(V_{GS_{BG}} - V_{th_{BG}}\right) V_{DS_{BG}} \tag{1}$$

where C_{SiO_2} is the SiO_2 capacitance per unit area, W and L are the transistor channel width and length, respectively, $V_{GS_{BG}}$ is the back gate voltage and $V_{DS_{BG}}$ is the drain voltage. Then, assuming that the field-effect mobility is equal for devices fabricated in BG and TG configuration and matching their drain current I_{DS}, we derived the value of the hBN capacitance using Equation 2:

$$C_{hBN} = C_{SiO_2} \frac{\left(V_{GS_{BG}} - V_{th_{BG}}\right) V_{DS_{BG}}}{\left(V_{GS_{TG}} - V_{th_{TG}}\right) V_{DS_{TG}}} \tag{2}$$

The average hBN capacitance value C_{hBN} is 760 nF/cm^2.

Table S2: Table summarizing key device parameters including off-current (I_{OFF}), on-current (I_{ON}), on/off ratio (I_{ON}/I_{OFF}), threshold voltage (V_{TH}), and subthreshold slope (SS).

	**MoS_2 based FETs with Gr contacts on SiO_2**				
	**I_{off} (μA)**	**I_{on} (μA)**	**I_{on}/I_{off}**	**V_{th} (V)**	**SS (mV/dec)**
Device 1	9.91×10^{-9}	8.98×10^{-7}	9.06×10^{1}	1.45	580
Device 2	1.19×10^{-8}	1.03×10^{-6}	8.64×10^{1}	2.01	525
Device 3	9.00×10^{-9}	1.45×10^{-6}	1.61×10^{2}	2.08	490

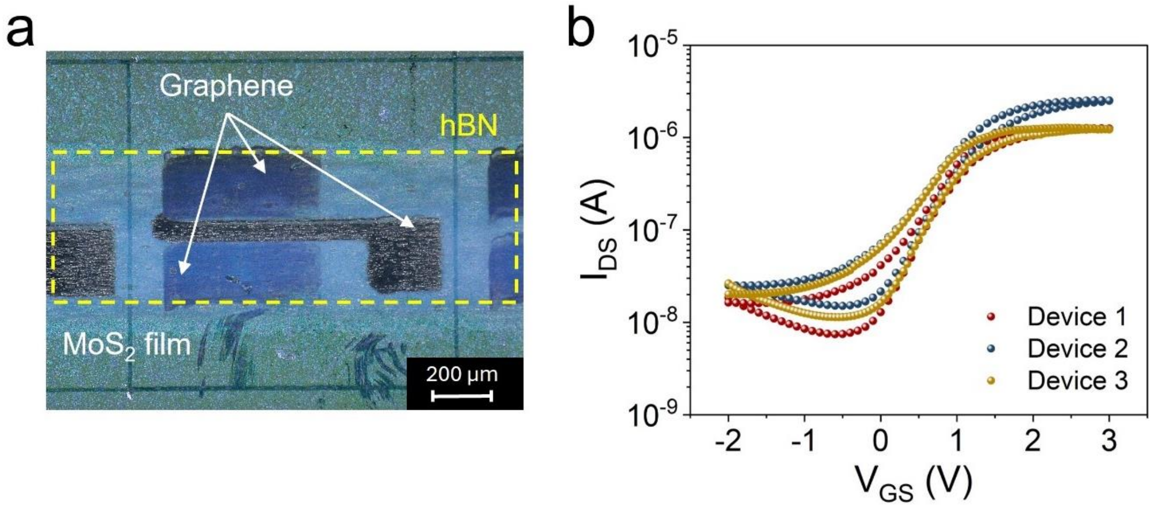

Figure S11: (a) Optical microscopic images of FETs with printed graphene S/D and gate contacts on silicon. (b) Transfer characteristics showing drain current (I_{DS}) vs. gate voltage (V_{GS}) curves for all devices with Gr contacts, demonstrating consistent switching behaviour with slight variations in performance metrics.

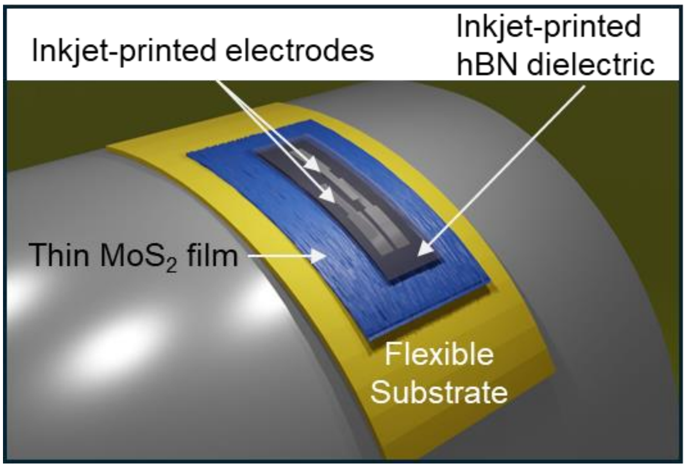

Figure S12: Schematic representation of the transistor with inkjet printed (Ag or Gr) electrodes, hBN dielectric, and MoS_2 as semiconductor on a flexible polyimide (PI) substrate

Section 5. Electrical characterization on flexible substrate.

These electrical measurements present performance data for ten different MoS_2-based TFT with silver (Ag) contacts fabricated on flexible polyimide substrate. Looking at the transfer characteristics and extracted parameters, we observe higher subthreshold slope values (ranging from 350 to 580 mV dec^{-1}) compared to devices on rigid substrates. The threshold voltages remain relatively uniform, around 1.3-1.7 V. The overlapping transfer curves remark the reproducibility of the electrical characteristics across multiple devices on the flexible substrate.

Table S3: Table summarizing key device parameters including off-current (I_{OFF}), on-current (I_{ON}), on/off ratio (I_{ON}/I_{OFF}), threshold voltage (V_{TH}), and subthreshold slope (SS).

	MoS$_2$ based FETs on polyimide (PI) substrate				
	I_{off} (µA)	I_{on} (µA)	I_{on}/I_{off}	V_{th} (V)	SS (mV/dec)
Device 1	9.91×10^{-9}	8.98×10^{-7}	9.06×10^{1}	1.45	580
Device 2	1.19×10^{-8}	1.03×10^{-6}	8.64×10^{1}	1.61	525
Device 3	9.00×10^{-9}	1.45×10^{-6}	1.61×10^{2}	1.68	490
Device 4	8.89×10^{-9}	2.25×10^{-6}	2.53×10^{2}	1.32	420
Device 5	7.49×10^{-9}	1.51×10^{-6}	2.01×10^{2}	1.65	430
Device 6	9.91×10^{-9}	2.73×10^{-6}	2.76×10^{2}	1.39	430
Device 7	1.13×10^{-8}	2.45×10^{-6}	2.17×10^{2}	1.35	410
Device 8	1.56×10^{-8}	2.59×10^{-6}	1.66×10^{2}	1.32	460
Device 9	1.01×10^{-8}	2.91×10^{-6}	2.88×10^{2}	1.3	370
Device 10	8.66×10^{-9}	2.43×10^{-6}	2.81×10^{2}	1.21	350

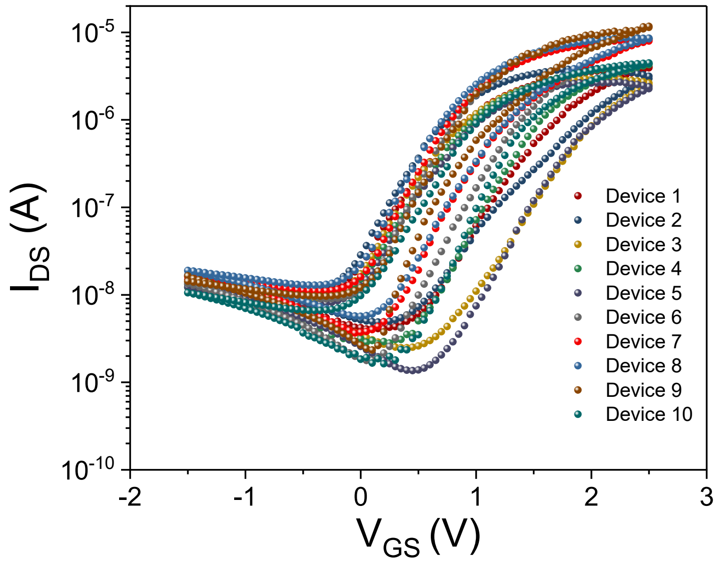

Figure S13: Transfer characteristics showing drain current (I_{DS}) vs. gate voltage (V_{GS}) curves for all devices, demonstrating consistent switching behaviour with slight variations in performance metrics. The devices exhibit typical n-type semiconductor behaviour with on/off ratios ranging from 8.64×10^{1} to 3.88×10^{2} and threshold voltages between 1.21 V and 1.68 V.

Table S4: Table summarizing key device parameters including off-current (I_{OFF}), on-current (I_{ON}), on/off ratio (I_{ON}/I_{OFF}), threshold voltage (V_{TH}), and subthreshold slope (SS).

	MoS₂ based FETs with Gr contacts on PI				
	**I_{off} (μA)**	**I_{on} (μA)**	**I_{on}/I_{off}**	**V_{th} (V)**	**SS (mV/dec)**
Device 1	9.91×10^{-9}	8.98×10^{-7}	9.06×10^{1}	1.45	580
Device 2	1.19×10^{-8}	1.03×10^{-6}	8.64×10^{1}	2.01	525
Device 3	9.00×10^{-9}	1.45×10^{-6}	1.61×10^{2}	2.08	490
Device 4	8.89×10^{-9}	2.25×10^{-6}	2.53×10^{2}	1.32	420

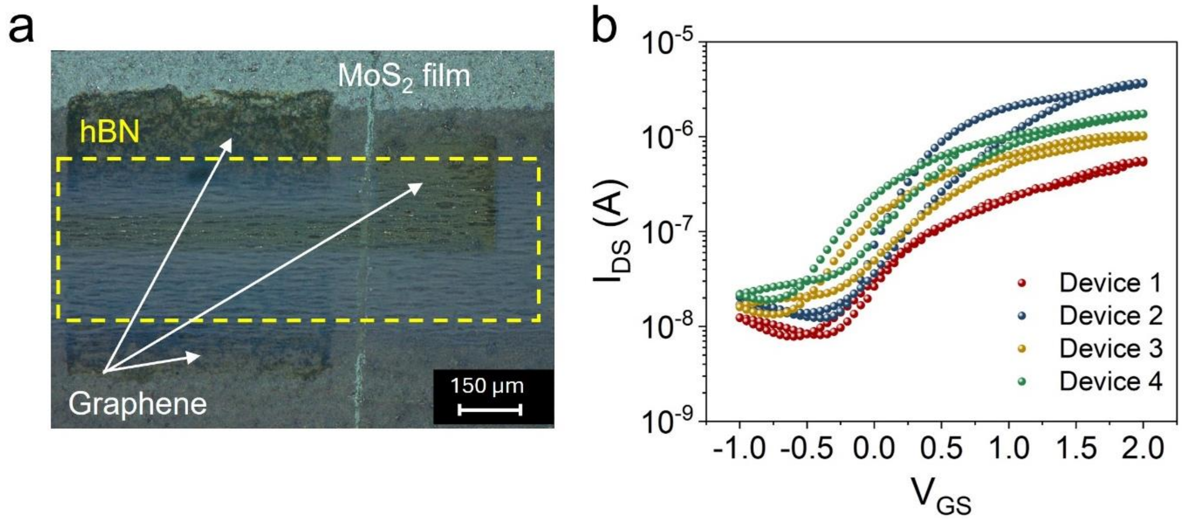

Figure S14: (a) Optical microscopic images of FETs with printed graphene S/D and gate contacts on polyimide (PI). (b) Transfer characteristics showing drain current (I_{DS}) vs. gate voltage (V_{GS}) curves for all devices with Gr contacts, demonstrating consistent switching behaviour with slight variations in performance metrics.

Supplementary Section 6. Electrical measurements under bending

The electrical characteristics of the MoS_2-based TFT under mechanical strain reveal robust device operation across various bending conditions. Starting from the unbent state, the device maintains its switching behaviour as the bending radius decreases from 4 cm to 2.5 cm. A systematic decrease in the maximum drain current is observed with increasing mechanical strain (decreasing bending radius), suggesting strain-induced changes in the electronic properties of the MoS_2 channel. When the device is returned to its original unbent state (purple curve), the electrical characteristics show substantial recovery, though with some residual hysteresis, indicating the largely reversible nature of the strain effects.

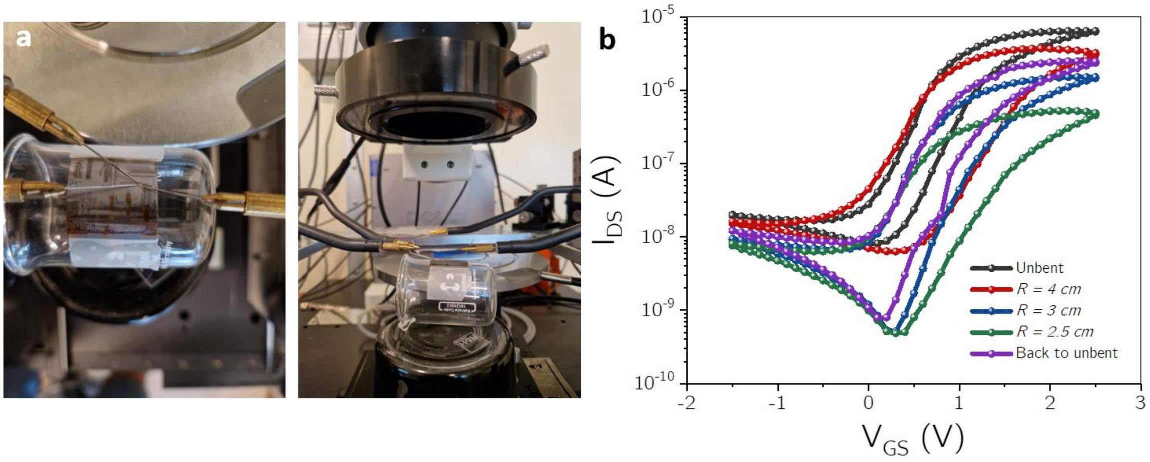

Figure S15: (a) Optical pictures showing the experimental setup for controlled mechanical bending of the device using cylinders with different radii. (b) Transfer characteristics measured at different bending radii (R), ranging from unbent state to R = 2.5 cm. The device demonstrates maintained semiconducting behaviour under various bending conditions, with the "Back to unbent" curve (purple dots) showing reasonable recovery of electrical properties after mechanical deformation, indicating the robustness of the flexible device structure.

Table S5: State-of-art comparison table showing solution processable 2D materials-based FETs reported in Figure 3f and 4f of the main text. The power supply (i.e. the range of the applied gate voltage) is classified as high voltage (HV, >3V) or low voltage (LV, ≤3V), G = gate electrode; S/D = source/drain electrodes, lin. (linear regime); sat. (saturation regime); BGBC = bottom-gate bottom-contact; TGBC = top-gate bottom-contact; TC = top-contact.

No. of solution processed components	Semiconductor synthesis/deposition technique/film thickness	Insulator deposition technique	Contacts deposition technique	Transistor geometry	Substrate	Post-processing	Measurement conditions	Capacitance (nF cm^{-2})	Power supply (V)	Mobility (cm^2 V^{-1} s^{-1})	I_{ON}/I_{OFF}	V_{th} (V)	Ref
**Rigid substrates**													
2	LPE MoS_2 / Inkjet printing / 50-250 nm	SiO_2 (300 nm)	Ag (S/D) Inkjet printing	BGBC	Si/SiO_2	Annealing at 450 °C in N_2	Ambient conditions	11.6	HV	2	28 (lin.)	--	[1]
2	LPE MoS_2 / Drop casting, functionalization with BDT in hexane/ 700nm	EMIm-TFSI	Au Thermal evaporation	BGBC	Si/SiO_2	N_2 glovebox for the following functionalization: 1. Sample immersion in 50 mM saturated solution of BDT in anhydrous hexane for 24 h inside a sealed container. 2. Spin rinsing with hexane 3. Annealing at 90 °C for 30-45 min	LV	--	LV	0.01	10^4 (lin.)	1.8±0.1	[2]
2	ECE MoS_2 in PVP-DMF/ L-b-L/7-30 nm	PEGDA/HOMPP/EMIm-TFSI Spin coating	Cr/Au (G/S/D)	IL-gated BC	Si/SiO_2	1. PDDA treatment	Vacuum/room temp.	6000	LV	9.8	1 × 10^5 (lin.)	--	[3]

			Thermal evaporation			2. TFSI treatment in 1,2-dichloroethane at 80 °C for 2 h<br>3. Thermal treatment at 300 °C							
3	N-graphene/ LPE MoS_2/ Inkjet printing/ 100-150 nm	$BaTiO_3$ nanoparticle/ Inkjet printing	Ag/ Inkjet printing	TGBC	Glass	The printed dielectric was first backed at 100 °C for 20 min and subsequently backed at 230 °C for 30 min	Ambient conditions	16.4	HV	0.01	11.4 (sat.)	--	4
3	ECE MoS_2/ Spin coating	HfO_2/ spin coating and oxidized from HfS_2 at 500 °C for 5 h	Graphene / spin coating	BGTC	Si/SiO_2	80 °C and TFSI treatment Annealing at 500 °C	Vacuum (≈10^{-5} Torr)	400-500	LV	8.3	10^5 (lin.)	--	5
			Graphene/ Inkjet printing (300 °C, 30 min)			200 °C in Ar 500 °C				4.0	10^2 (sat.)	--	
2	ECE MoS_2/ Inkjet printing / 18 nm	SiO_2 (300 nm)	Graphene/ Inkjet printing (300 °C, 30 min)	BGTC	Si/SiO_2	200 °C in Ar, 300 °C	Vacuum (≈10^{-5} Torr)	11.6	HV	0.02	7.5 × 10^3 (lin.)	--	6
3		HfO_2/ Inkjet printing and oxidized from HfS_2 at 500 °C for 5 h						436	LV	4	3.4 × 10^5 (sat.)	1.5	
2	ECE MoS_2 in PVP-DMF/ Langmuir-Scheafer deposition/ 14 nm	EMIm-TFSI/ drop casting	Au (S/D)/ Thermal evaporation	IL-gated/ TC	Si/SiO_2	120 °C in N_2, 1 h	Ambient conditions	3100	LV	10.7	2 × 10^3	0.38±0.05	7
	ECE WSe_2 in PVP-DMF/									2	4 x 10^3	1.63±0.01	

	Langmuir-Schaefer/ 14 nm												
	ECE WS_2 in PVP-DMF/ Langmuir-Schaefer/ 10.5 nm									9.1	2 × 10^3	0.33±0.09	
2	LPE WS_2/ Spray coating/ 1000 nm	EMIm-TFSI/ drop casting	Cr/Au (S/D)/ e-beam evaporation	IL-gated/ TC	Glass	120 °C	N_2/ vacuum (<10^{-6} mbar)	3.5 x 10^7	LV	0.013	10^4 (sat.)	1.0±0.1	8
3	ECE MoS_2 in PVP-Chloroform/ spin coating/ 12 nm	HfO_2/ spin coating of HfS_2-azide crosslinker, UV exposure and oxidized from HfS_2 at 500 °C for 5 h in air	Graphene/spin coating/ UV exposure to azide crosslinker and annealing at 300 °C for 30 min in Ar glovebox	BGTC	Si/SiO_2	TFSI treatment Annealing at 250 °C 30 min Final annealing at 300 °C for 30 min in Ar glovebox	Vacuum (≈10^{-4} Torr)	260.4	HV	20.3	1.36 × 10^6	1.6	9
2	ECE MoS_2 in PVP-IPA/ Slot-die coating/ 15 nm	Na-embedded alumina/ Slot-die coating/ 500 °C 2h	Cr/Au (G/S/D) Thermal evaporation	BGTC	Si/SiO_2 Or ITO/glass	TFSI treatment Annealing at 250 °C 30 min	Vacuum (≈10^{-4} Torr)	840±70	LV	80	5 × 10^4	3.23	10
2	ECE MoS_2 in PVP-IPA/ Spin coating/ 7 nm	$Sr_{1.8}Bi_{0.2}Nb_3O_{10}$/ Langmuir-Blodgett assembly/ 110 °C for 30 min; UV irradiation 24 h	Ti/ Au Thermal evaporation	TGTC	P^{++} Si	TFSI treatment Annealing at 250 °C 1 h	Ambient conditions	0.03	LV	11	10^6	--	11
2	ECE MoS_2 in PVP-IPA/ Langmuir-Schaefer / 15 nm	EMIm-TFSI/ drop casting	Ti/ Au Thermal evaporation	TGTC	Glass	Annealing at 120 °C in Ar for 1 h	Ambient conditions	2000	LV	6.6	10^3	--	12
2	Thermolysis of $(NH_4)_2MoS_4$ to to grow MoS_2 film / Electrohydrodyna	SiO_2 (300 nm)	Ag/ EHD printing	BGTC	Si/SiO_2	Annealing at 1000 °C for 1 h for MoS_2 growth	Ambient conditions	--	HV	0.07 (Avg.)	1 × 10^4	--	13

	mic jet printing (EHD)					Annealing 150 °C for 30 min							
**Flexible substrates**													
2	ECE MoS_2 in PVP-DMF/ L-b-L/7-30 nm	PEGDA/HOMPP/EMIm-TFSI Spin coating	Cr/Au (G/S/D) Thermal evaporation	IL-gated BC	PET	1. PDDA treatment 2. TFSI treatment in 1,2-dichloroethane at 80 °C for 2 h 3. Annealing 2h in Ar	Vacuum/ room temp.	6000	LV	0.5	$>10^4$	--	3
2	ECE WSe_2 in PVP-DMF/ Langmuir-Schaefer/ 14 nm	EMIm-TFSI/ drop casting	Au (S/D)/ Thermal evaporation	IL-gated/ TC	PET	120 °C in N_2, 1 h	a.c.	3100	LV	1.9	2.9×10^3	1.63±0.01	7
2	LPE WSe_2/ Spray coating/ 1500 nm	Graphene (side gate)/ Inkjet printing EMIm-TFSI/ drop casting	Au (S/D)/ Thermal evaporation	IL-gated/ TC	Alumina coated PET	70 °C, 10^{-5} mbar, 12 h	High vacuum	--	LV	0.025	$\sim10^3$	--	14
2	LPE MoS_2/ Spray coating/ 1000 nm	EMIm-TFSI/ drop casting	Au (S/D)/ Thermal evaporation	IL-gated/ TC	Alumina coated PET	70 °C, 10^{-5} mbar, 12 h	High vacuum	2×10^5	LV	0.15	$<10^2$ (sat.)	0.2	15
	LPE $MoSe_2$/ Spray coating/ 1100 nm							2×10^5		0.18	$<10^2$ (sat.)	0.9	
	LPE WS_2/ Spray coating/ 1800 nm							1.95×10^5		0.22	3×10^2 (sat.)	1.5	
	LPE WSe_2/ Spray coating/ 950 nm							1.2×10^5		0.08	$<10^2$ (sat.)	≈0.55	
3	LPE WS_2/ Spray coating/ 1000 nm	EMIm-TFSI/ h-BN spray coating	Graphene/ Inkjet printing	h-BN with IL-gate				--		--	25 (sat.)	--	
2	LPE WSe_2/ Aerosol Jet printing/ 3000 nm	EMIm-TFSI/ drop casting	Graphene/ Inkjet printing	IL-gated/ TC	Alumina coated PET	80 °C	High vacuum	--	LV	1.5×10^{-3}	20 (lin.)	--	16
2	LPE WS_2/graphene/ Spray coating/ 1000 nm	EMIm-TFSI/ drop casting	Cr/Au (S/D/G)/ Thermal evaporation	IL-gated/ TC	Alumina coated PET	70 °C, 10^{-5} mbar, 12 h	High vacuum	--	LV	0.3	10^4 (sat.)	≈0.5	17

**3**	**ECE MoS_2/ liquid-liquid interfacial assembly (LLI)/ 60 nm**	**h-BN / Inkjet printing**	**Ag (S/D) / Inkjet printing**	**TGTC**	**Si/SiO_2**	**300 °C for 2 h in vacuum**	**Ambient/ room temp.**	**760**	**LV**	**3.16 (avg.) 6.29 (best)**	**3.04 × 10^3**	**1.5**	**This work**
			**Graphene (S/D) / Inkjet printing**							**0.19 (avg.) 0.23 (best)**	**1.1× 10^2**	**0.8**	
			**Ag (S/D) / Inkjet printing**		**Polyimide (PI)**					**0.98 (avg.) 2.47 (best)**	**2.02 × 10^2**	**1.4**	
			**Graphene (S/D) / Inkjet printing**							**0.23 (avg.) 0.46 (best)**	**1.3 × 10^2**	**0.4**	

References:

1. Li, J., Naiini, M. M., Vaziri, S., Lemme, M. C. & Östling, M. "Inkjet Printing of MoS2". *Adv. Funct. Mater.* 24 (2014): 6524-6531.
2. Ippolito, S., Kelly, A. G., Furlan de Oliveira, R. *et al.* "Covalently interconnected transition metal dichalcogenide networks via defect engineering for high-performance electronic devices". *Nat. Nanotechnol.* 16 (2021): 592-598.
3. Gao, X., Yin, J., Bian, G. *et al.* "High-mobility patternable MoS2 percolating nanofilms". *Nano Res.* 14 (2021): 2255-2263.
4. Jewel, M. U., Monne, M. A., Mishra, B. & Chen, M. Y. "Inkjet-Printed Molybdenum Disulfide and Nitrogen-Doped Graphene Active Layer High On/Off Ratio Transistors". *Molecules* 25(2020): 1081
5. Kim, J., Rhee, D., Song, O. *et al.* "All-Solution-Processed Van der Waals Heterostructures for Wafer-Scale Electronics". *Adv. Mater.* 34 (2022): 2106110.
6. Song, O., Rhee, D., Kim, J. *et al.* "All inkjet-printed electronics based on electrochemically exfoliated two-dimensional metal, semiconductor, and dielectric". *npj 2D Mater. Appl.* 6 (2022): 64.
7. Carey, T., Cassidy, O., Synnatschke, K. *et al.* "High-Mobility Flexible Transistors with Low-Temperature Solution-Processed Tungsten Dichalcogenides". *ACS Nano* 17 (2023): 2912-2922.
8. Higgins, T. M., Finn, S., Matthiesen, M. *et al.* "Electrolyte-Gated n-Type Transistors Produced from Aqueous Inks of WS2 Nanosheets". *Adv. Funct. Mater.* 29 (2019): 1804387.
9. Kwak, I. C., Kim, J., Moon, J. W. *et al.* "Orthogonal photopatterning of two-dimensional percolated network films for wafer-scale heterostructures". *Nat. Electron.* 8 (2025): 235-243.
10. Kwon, Y. A., Kim, J., Jo, S. B. *et al.* "Wafer-scale transistor arrays fabricated using slot-die printing of molybdenum disulfide and sodium-embedded alumina". *Nat. Electron.* 6 (2023): 443-450.
11. Joung, S.-Y., Yim, H., Lee, D. *et al.* "All-Solution-Processed High-Performance MoS2 Thin-Film Transistors with a Quasi-2D Perovskite Oxide Dielectric". *ACS Nano* 18 (2024): 1958-1968.
12. Gabbett, C., Kelly, A. G., Coleman, E. *et al.* "Understanding how junction resistances impact the conduction mechanism in nano-networks". *Nat. Commun.* 15 (2024): 4517.
13. Can, T. T. T. & Choi, W.-S. "Stacked printed MoS2 and Ag electrodes using electrohydrodynamic jet printing for thin-film transistors". *Sci Rep.* 12 (2022): 22469.
14. O'Suilleabhain, D., Kelly, A. G., Tian, R. *et al.* "Effect of the Gate Volume on the Performance of Printed Nanosheet Network-Based Transistors". *ACS Appl. Electron. Mater.* 2 (2020): 2164-2170.
15. Kelly, A. G., Hallam, T., Backes, C. *et al.* "All-printed thin-film transistors from networks of liquid-exfoliated nanosheets". *Science* 356 (2017): 69-73.
16. Kelly, A. G., Vega-Mayoral, V., Boland, J. B. & Coleman, J. N. "Whiskey-phase exfoliation: exfoliation and printing of nanosheets using Irish whiskey". *2D Mater.* 6 (2019): 045036.

17 O'Suilleabhain, D., Vega-Mayoral, V., Kelly, A. G., Harvey, A. & Coleman, J. N. "Percolation Effects in Electrolytically Gated WS2/Graphene Nano:Nano Composites". *Acs Appl Mater Inter* 11 (2019): 8545-8555.
